# Reading between the Lines: Utilizing RNA-Seq Data for Global Analysis of sRNAs in Staphylococcus aureus

**DOI:** 10.1128/mSphere.00439-20

**Published:** 2020-07-29

**Authors:** Hailee M. Sorensen, Rebecca A. Keogh, Marcus A. Wittekind, Andrew R. Caillet, Richard E. Wiemels, Elizabeth A. Laner, Ronan K. Carroll

**Affiliations:** a Department of Biological Sciences, Ohio University, Athens, Ohio, USA; b Honors Tutorial College, Ohio University, Athens, Ohio, USA; c Infectious and Tropical Disease Institute, Ohio University, Athens, Ohio, USA; University of Nebraska Medical Center

**Keywords:** RNA stability, RNA-Seq, *Staphylococcus aureus*, genome annotation, regulatory RNA, sRNA, small peptides

## Abstract

Regulatory small RNAs (sRNAs) are known to play important roles in the Gram-positive bacterial pathogen Staphylococcus aureus; however, their existence is often overlooked, primarily because sRNA genes are absent from genome annotation files. Consequently, transcriptome sequencing (RNA-Seq)-based experimental approaches, performed using standard genome annotation files as a reference, have likely overlooked data for sRNAs. Previously, we created an updated S. aureus genome annotation file, which included annotations for 303 known sRNAs in USA300. Here, we utilized this updated reference file to reexamine publicly available RNA-Seq data sets in an attempt to recover lost information on sRNA expression, stability, and potential to encode peptides. First, we used transcriptomic data from 22 studies to identify how the expression of 303 sRNAs changed under 64 different experimental conditions. Next, we used RNA-Seq data from an RNA stability assay to identify highly stable/unstable sRNAs. We went on to reanalyze a ribosome profiling (Ribo-seq) data set to identify sRNAs that have the potential to encode peptides and to experimentally confirm the presence of three of these peptides in the USA300 background. Interestingly, one of these sRNAs/peptides, encoded at the *tsr37* locus, influences the ability of S. aureus cells to autoaggregate. Finally, we reexamined two recently published *in vivo* RNA-Seq data sets, from the cystic fibrosis (CF) lung and a murine vaginal colonization study, and identified 29 sRNAs that may play a role *in vivo*. Collectively, these results can help inform future studies of these important regulatory elements in S. aureus and highlight the need for ongoing curating and updating of genome annotation files.

**IMPORTANCE** Regulatory small RNAs (sRNAs) are a class of RNA molecules that are produced in bacterial cells but that typically do not encode proteins. Instead, they perform a variety of critical functions within the cell as RNA. Most bacterial genomes do not include annotations for sRNA genes, and any type of analysis that is performed using a bacterial genome as a reference will therefore overlook data for sRNAs. In this study, we reexamined hundreds of previously generated S. aureus RNA-Seq data sets and reanalyzed them to generate data for sRNAs. To do so, we utilized an updated S. aureus genome annotation file, previously generated by our group, which contains annotations for 303 sRNAs. The data generated (which were previously discarded) shed new light on sRNAs in S. aureus, most of which are unstudied, and highlight certain sRNAs that are likely to play important roles in the cell.

## Abstract (precis)

Regulatory small RNAs (sRNAs) are a class of RNA molecules that are produced in bacterial cells but that typically do not encode proteins. Instead, they perform a variety of critical functions within the cell as RNA. Most bacterial genomes do not include annotations for sRNA genes, and any type of analysis that is performed using a bacterial genome as a reference will therefore overlook data for sRNAs. In this study, we reexamined hundreds of previously generated S. aureus RNA-Seq data sets and reanalyzed them to generate data for sRNAs. To do so, we utilized an updated S. aureus genome annotation file, previously generated by our group, which contains annotations for 303 sRNAs. The data generated (which were previously discarded) shed new light on sRNAs in S. aureus, most of which are unstudied, and highlight certain sRNAs that are likely to play important roles in the cell.

## INTRODUCTION

In bacterial cells, the best-studied and most abundant RNA molecules (rRNA, tRNA, and mRNA) play prominent roles in protein synthesis. Members of a less-well-studied class of RNA molecules that perform a variety of functions in the cell are collectively referred to as small RNAs (sRNAs). Many of these sRNAs are regulatory RNAs that influence gene expression by interacting with mRNA transcripts and/or proteins (for a review of bacterial sRNAs, see reference 1). Although sRNAs play important roles in the cell, their study has lagged behind those of protein coding genes for a variety of reasons. One reason is that sRNA genes are difficult to identify by sequence analysis alone. Another factor confounding the study of sRNAs is the fact that annotations for sRNA genes are typically absent from genome annotation files. This has led to repeat identifications of sRNAs and a general lack of awareness of the genomic location of sRNA genes (2). Furthermore, as genome annotation files are used as a reference for transcriptome sequencing (RNA-Seq) studies, the resulting analyses typically do not contain expression values for sRNAs because sRNA genes are absent from these files.

In the Gram-positive bacterium Staphylococcus aureus, hundreds of sRNA molecules have been identified (3). To facilitate further analysis of sRNAs in this important human pathogen, we previously performed a study whereby we annotated the position of known sRNAs on the S. aureus genome (2). The resulting annotation files (created in three S. aureus genetic backgrounds) are a valuable resource and have allowed us to (i) identify new sRNAs (with confidence that they had not been already identified), and (ii) analyze global sRNA gene expression by RNA-Seq (2). Recently, we further demonstrated the benefit of these annotation files by investigating the role of an sRNA, Teg41, that is in a location adjacent to the genes encoding the alpha phenol-soluble modulins (αPSMs), potent cytolytic peptides produced by S. aureus (4). Our study revealed that αPSM peptide production is positively influenced by the Teg41 sRNA (4).

During our sRNA annotation study, we included annotations for 303 sRNAs on the genome of the USA300 strain FPR3757, 39 of which we identified in the previous study (2). For clarity, as outlined in the annotation study, we used the term “sRNA” to refer to any RNA that had not been previously annotated (including riboswitches, untranslated regions [UTRs], and *cis*-acting and *trans-*acting sRNAs). The rationale behind this was to provide us with a more informative map of the S. aureus genome in which the locations of key RNA features were more apparent and to generate a tool that could be used for further studies. Undoubtedly, the list of “sRNAs” that we annotated is imperfect. sRNAs that are expressed only under specific conditions (not thus far tested) are likely to be missing from the list. Furthermore, it is also likely that some of the annotations added do not represent genuine sRNAs. As mentioned above, we did not restrict our annotation to *trans*-acting sRNAs; therefore, many of the annotated “sRNA genes” are riboswitches, UTRs, and *cis-*acting elements. Furthermore, we consider it likely that many of the annotations added in fact represent protein coding genes. Consistent with this idea, we noted that certain loci where sRNA annotations were added in USA300 FPR3757 correspond to loci where coding DNA sequence (CDS) genes had been annotated in other S. aureus strains (2). These may represent protein coding genes that were omitted from the USA300 FPR3757 annotation. Consequently, while we acknowledge that the created annotation files are imperfect, they are nonetheless a valuable resource and represent a first step toward a fully annotated S. aureus genome.

As mentioned above, the absence of sRNA annotations in genome reference files has meant that all previously published RNA-Seq studies in S. aureus have failed to analyze sRNAs. Therefore, in this study, we revisited publicly available RNA-Seq data sets deposited in the Gene Expression Omnibus (GEO) database, to mine them for sRNA data. Using our annotated sRNA genome file as a reference, we examine (i) RNA-Seq transcriptomic data sets to analyze sRNA gene expression levels in a variety of strain backgrounds and under a variety of environmental conditions; (ii) RNA-Seq data from rifampin-treated cultures to analyze the rates of sRNA decay and stability; (iii) a ribosome profiling (Ribo-seq) data set to determine which of the 303 previously annotated sRNAs are bound by ribosomes and therefore potentially encode peptides; and (iv) two recently published *in vivo* RNA-Seq studies to search for sRNAs that are upregulated during infection.

In 2016, Mäder et al. performed a comprehensive analysis of S. aureus gene expression using tiling microarrays (5). The results generated provide an analysis of gene expression in an isogenic S. aureus background, across a variety of conditions, and represent an immensely valuable research tool. The study also identified transcriptional units that contain sRNAs, examined their expression, and compared expression to that of protein coding genes. Results from that analysis also suggest that the number of *trans-*acting sRNAs in S. aureus is lower than previous studies had suggested. A recent publication by Liu et al. supports with this assessment and suggests that the number of bona fide *trans-*acting sRNAs in S. aureus is around 50 (6).

Unlike the study by Mäder et al., the results presented here do not represent a comprehensive global transcriptomic analysis of isogenic strains across different growth media. Consequently, we have restricted our analysis to compare only RNA-Seq data sets from studies performed by the same research group from projects submitted to GEO. This ensures that all comparisons are performed in the same S. aureus genetic background. We also perform the analysis using the 303 “sRNA annotations” added during our 2016 study while acknowledging the imperfect nature of this list. To validate our approach, we confirmed the stability profiles of two sRNAs by Northern blotting. We also demonstrate, on the basis of the results from the Ribo-seq data set, that a number of previously identified sRNAs have the ability to encode short peptides/proteins and show that overproduction of one such peptide (Tsr37) in S. aureus led to increased “clumping” of the bacterial cells (i.e., rapid autoaggregation and settling of the cells at the bottom of the culture tube), which may indicate a potential biological role. While imperfect, these results were generated using freely available, previously discarded data and can serve as a valuable guide to inform future studies and facilitate improved analysis of sRNAs in S. aureus.

## RESULTS AND DISCUSSION

### Overview of study.

**FIG 1:**
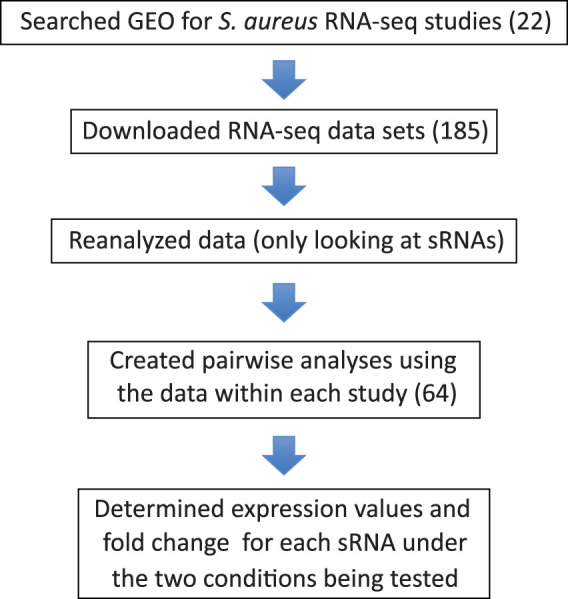
Overall design of global sRNA expression study. Numbers in parentheses represent numbers of studies, data sets, or analyses as indicated.

Due to the fact that sRNA annotations are missing from S. aureus genome files, we reasoned (i) that all previously published RNA-Seq data sets/experiments from S. aureus would have generated reads corresponding to sRNAs but (ii) that such data would have been discarded during the data analysis step. Consequently, a reanalysis of existing data sets performed using our sRNA annotated genome files would likely give information on the expression of sRNAs that had previously been missed. A flowchart outlining the reanalysis procedure is provided in Fig. 1. We searched the Gene Expression Omnibus (GEO) online data repository for RNA-Seq data sets originating from S. aureus (see Text S1, S2, and S3 in the supplemental material for an overview of the criteria used to search for data sets). Through this search, we identified 185 individual RNA-Seq data sets from 22 studies for further analysis (Table 1; see also Data Set S1 in the supplemental material).

**TABLE 1:** 22 studies used for global sRNA expression analysis^*a*^

Study	GEO project title	SRA ID	Total no. of datasets	Total no. of datasets used in this study	Reference
001	Response of *nor* and *nos* contributes to *Staphylococcus aureus* virulence and metabolism	SRP144815	30	30	NA
002	Characterization of the LFR Genomic Islet in *Staphylococcus aureus* CC30	SRP077878	14	14	NA
003	Changes in relative transcript amounts resulting from hydrogen sulfide treatment, calprotectin treatment, and deletion of CstR in *Staphylococcus aureus*	SRP108274	18	18	19
004	RNASeq analysis of untreated versus treated	SRP166268	6	6	20
005	A master virulence regulator of *S. aureus* inactivated during carriage in man	SRP056736	12	12	21
006	RNA-Seq-mediated transcriptome analysis of *Staphylococcus aureus* Newman wild-type, walKD119A, walKV149A and DHBP-treated wild-type strains	SRP067051	8	8	22
007	The conserved regulatory RNA RsaE down-regulates the arginine degradation pathway in *Staphylococcus aureus* [ssRNA-Seq]	SRP123506	10	10	23
008	Changes in relative transcript amounts caused by treatment of streptozotocin and floxuridine in *S. aureus* USA300	SRP118369	9	9	24
009	Effects of fosfomycin on biofilm of a clinical *Staphylococcus aureus* isolated from osteoarticular infection by transcriptomal approach	SRP076463	12	12	7
010	Linoleic Acid Stimulation of WT *Staphylococcus aureus* USA300 NRS384	SRP114855	6	6	25
011	RNA-Seq of Wildtype *Staphylococcus aureus* USA300 NRS384	SRP114862	6	6	25
012	RNA-Seq based comparison of *Staphylococcus aureus* strains resistant and sensitive to MT02	SRP090765	4	4	26
013	The *Staphylococcus aureus* α-Acetolactate Synthase ALS Confers Resistance to Nitrosative Stress	SRP108521	4	4	27
014	Response to low-level phage predation in *Staphylococcus aureus* biofilms	SRP090939	6	6	28
015	Phenotype and RNA-Seq-Based Transcriptome Profiling of *Staphylococcus aureus* biofilms in response to Tea tree oil	SRP082322	4	4	NA
016	Comparative virulence studies and transcriptome analysis of *Staphylococcus aureus* strains isolated from animals	SRP070956	4	4	29
017	Decay-initiating endoribonucleolytic cleavage by RNase Y is kept under tight control via sequence preference and sub-cellular localisation	SRP058261	24	4	30
018	Next Generation Sequencing Facilitates Quantitative Analysis of Wild Type and △Rsp MRSA Transcriptomes	SRP056619	4	4	31
019	RNA-SEQ Reveals Changes in the *Staphylococcus aureus* Transcriptome following blue light illumination	SRP048638	4	4	32
020	The C-terminal region of the RNA helicase CshA is required for the interaction with the degradosome and turnover of bulk RNA in the opportunistic pathogen *Staphylococcus aureus*	SRP058249	24	6	11
021	SaeRS-dependent inhibition of biofilm formation in *Staphylococcus aureus* Newman	SRP053597	10	10	33
022	Steady-state hydrogen peroxide induces glycolysis via metabolic reroute in *P. aeruginosa* and *S. aureus*	SRP039358	8	4	34

a DHBP, 2,4-dihydroxybenzophenone; ID, identifier; NA, not applicable; MRSA, methicillin-resistant Staphylococcus aureus; *nor*, nitric oxide reductase gene; *nos*, nitric oxide synthase gene; ssRNA, single-stranded RNA; WT, wild type.

10.1128/mSphere.00439-20.1 TEXT S1 Supplemental materials and methods. Download Text S1, DOCX file, 0.1 MB. Copyright © 2020 Sorensen et al. 2020 Sorensen et al. This content is distributed under the terms of the Creative Commons Attribution 4.0 International license.

10.1128/mSphere.00439-20.2 TEXT S2 GEO Search results obtained using the term “Staphylococcus aureus RNAseq.” Download Text S2, DOCX file, 0.03 MB. Copyright © 2020 Sorensen et al. 2020 Sorensen et al. This content is distributed under the terms of the Creative Commons Attribution 4.0 International license.

10.1128/mSphere.00439-20.3 TEXT S3 GEO Search results obtained using the term “Staphylococcus aureus RNA-seq.” Download Text S3, DOCX file, 0.02 MB. Copyright © 2020 Sorensen et al. 2020 Sorensen et al. This content is distributed under the terms of the Creative Commons Attribution 4.0 International license.

10.1128/mSphere.00439-20.4 DATA SET S1 Data sets deposited in GEO from the 22 studies used in this analysis. Download Data Set S1, XLSX file, 0.1 MB. Copyright © 2020 Sorensen et al. 2020 Sorensen et al. This content is distributed under the terms of the Creative Commons Attribution 4.0 International license.

The data sets identified were generated in a variety of different S. aureus backgrounds and included data determined under different growth conditions as well as from a variety of wild-type and mutant strains. To streamline the analysis and generate results that could be easily interpreted, we elected to analyze all data sets using the same reference genome (USA300 FPR3757) and to perform only direct comparisons of sRNA gene expression between data sets generated in the same study (and therefore within the same genetic background). We hypothesized that the degree of sequence similarity between sRNAs from different S. aureus backgrounds would be sufficiently high that RNA-Seq reads generated in one strain (e.g., strain Newman) would map to the corresponding sRNA gene on a different S. aureus reference genome (i.e., USA300 FPR3757). Therefore, we elected to analyze all data sets using the USA300 FPR3757 genome (to which we previously added sRNA annotations [2]). The resulting data generated represent a series of pairwise expression analyses rather than a systematic analysis of sRNA expression in a variety of strains and under a variety of conditions. We acknowledge the imperfect nature of this analysis; however, the goal was to generate an overview of sRNA expression under various conditions using already available data and the results generated are meant to serve as a guide to inform future studies.

### Data acquisition and global sRNA expression analysis.

From the 22 studies identified, 185 RNA-Seq data sets were downloaded from GEO and processed to calculate expression values for each of the 303 annotated sRNAs (Fig. 1). We next examined the conditions under which each experiment was performed to identify pairwise analyses that could be made using the data within each study. Only a single pairwise analysis was possible from the data within a certain study in some cases (e.g., studies 4, 10, and 11), while multiple pairwise analyses could be made in others (e.g., 13 pairwise analyses were performed using the 30 RNA-Seq data sets from study 1). In total, 64 pairwise analyses were performed from the 22 studies utilized. A complete list of the pairwise comparisons resulting from each study is provided in Table 2. For each of the 64 pairwise analyses performed, we determined (i) the expression values for each of the 303 annotated sRNAs under the two conditions being tested and (ii) the fold change in expression. The results obtained for each sRNA in each of the 64 pairwise analyses performed are provided as Data Set S2.

**TABLE 2:** 64 pairwise analyses^*a*^

Study	Pairwise comparison	Expt no.	Details	No. of sRNAs altered^*b*^
001	1	001.1	UAMS-1 anaerobic 0 min vs *nor* KO 0 min	5
	2	001.2	UAMS-1 anaerobic 120 min vs *nor* KO 120 min	9
	3	001.3	UAMS-1 anaerobic 240 min vs *nor* KO 240 min	14
	4	001.4	UAMS-1 anaerobic 0 min vs *nos* KO 0 min	68
	5	001.5	UAMS-1 anaerobic 120 min vs *nos* KO 120 min	102
	6	001.6	UAMS-1 anaerobic 240 min vs *nos* KO 240 min	3
	7	001.7	UAMS-1 0 mM DEA NONOate vs WT 2mM DEA NONOate	11
	8	001.8	UAMS-1 0 mM DEA NONOate vs *nor* KO 0 mM	9
	9	001.9	UAMS-1 0 mM DEA NONOate vs *nos* KO 0 mM	4
	10	001.10	UAMS-1 2 mM DEA NONOate vs *nor* KO 2 mM DEA NONOate	12
	11	001.11	UAMS-1 2 mM DEA NONOate vs *nos* KO 2 mM DEA NONOate	26
	12	001.12	UAMS-1 anaerobic 0 min vs UAMS-1 anaerobic 120 min	8
	13	001.13	UAMS-1 anaerobic 0 min vs UAMS-1 anaerobic 240 min	18

002	14	002.1	UAMS-1 ES vs delta *fad*	3
	15	002.2	UAMS-1 ES vs delta *mocR*	12
	16	002.3	UAMS-1 ES vs UAMS-1 LE	26
	17	002.4	UAMS-1 ES vs UAMS-1 EE	34
	18	002.5	UAMS-1 LE vs WT EE	15
	19	002.6	UAMS-1 LE vs delta *mocR*	7
	20	002.7	UAMS-1 EE vs delta *mocR*	3

003	21	003.1	Newman vs sulfide treated	15
	22	003.2	Newman vs delta *cstR*	44
	23	003.3	Newman vs nitroxyl treated	66
	24	003.4	Newman vs calprotectin treated	77

004	25	004.1	USA300 vs PBT2 and Zinc treated	15

005	26	005.1	JE2 vs NE13041	12
	27	005.2	JE2 vs patient P nasal	64
	28	005.3	JE2 vs patient P blood	69
	29	005.4	JE2 vs patient S nasal	73
	30	005.5	JE2 vs patient S blood	73
	31	005.6	Patient P nasal vs patient P blood	17
	32	005.7	Patient S nasal vs patient S blood	7

006	33	006.1	Newman vs *walK D119A* mutant	50
	34	006.2	Newman vs *walK V149A* mutant	51
	35	006.3	Newman vs DHBP treated	15

007	36	007.1	HG003 vs *rsaE* mutant	6
	37	007.2	HG003 vs *rsaE* plasmid complementation uninduced	57
	38	007.3	HG003 vs *rsaE* plasmid complementation induced with aTc	52
	39	007.4	*rsaE* mutant vs *rsaE* plasmid complementation uninduced	70
	40	007.5	*rsaE* mutant vs *rsaE* plasmid complementation induced with aTc	55
	41	007.6	*rsaE* plasmid complementation uninduced vs induced with aTc	6

008	42	008.1	USA300 vs floxuridine treated	85
	43	008.2	USA300 vs streptozotocin treated	37

009	44	009.1	LYO-S2 4-h biofilm no treatment vs 4-h biofilm fosfomycin treatment	52
	45	009.2	LYO-S2 24-h biofilm no treatment vs 24-h biofilm fosfomycin treatment	127
	46	009.3	LYO-S2 4-h biofilm no treatment vs 24-h biofilm no treatment	53
	47	009.4	4-h biofilm fosfomycin treated vs 24-h biofilm fosfomycin treated	103

010	48	010.1	USA300 untreated vs 10 μM linoleic acid treated	19

011	49	011.1	USA300 30°C vs 37°C	13

012	50	012.1	JE2 MT02-sensitive treated with MT02 vs JE2 MT02-resistant treated with MT02	17

013	51	013.1	USA300 untreated vs treated with spermine NONOate	58

014	52	014.1	IPLA 1 biofilm vs treated with phage phiIPLA-RODI	78

015	53	015.1	ATCC 29213 biofilm untreated vs biofilm treated with TTO	16

016	54	016.1	MRSA1679a vs ATCC 29213	57

017	55	017.1	PR01 vs RNase Y deletion	52

018	56	018.1	MRSA BD02-25 vs delta *rsp*	42

019	57	019.1	BUSA2288 blue light treatment vs no light treatment	29

020	58	020.1	PR01 0-s decay vs C-terminal deletion of *cshA* 0-s decay	27
020	59	020.2	PR01 0-s decay vs delta *cshA* 0-s decay	18
	60	020.3	C-terminal deletion of *cshA* 0-s decay vs delta *cshA* 0-s decay	32

021	61	021.1	Newman vs delta *saePQRS*	23
	62	021.2	Newman vs *saeS* repaired	18
	63	021.3	Delta *saePQRS* deletion vs *saeS* repaired	31

022	64	022.1	Newman untreated vs 10 mM hydrogen peroxide treated	18

a KO, knockout; *nor*, nitric oxide reductase gene; *nos*, nitric oxide synthase gene; DEA NONOate, nitric oxide donor diethylamine NONOate; ES, early stationary; LE, late exponential; EE, early exponential; *fad*, fatty acid desaturase gene; *mocR*, *mocR* regulator gene; *cstR*, CsoR-like sulfurtransferase repressor gene; PBT2, zinc ionophore; *walK*, sensor kinase of WalKR two-component system gene; DHBP, 2,4-dihydroxybenzophenone; aTc, anhydrotetracycline; floxuridine, chemotherapy drug (liver cancer); streptozotocin, chemotherapy drug (pancreatic cancer); fosfomycin, cell wall synthesis inhibiting antibiotic; linoleic acid, polyunsaturated omega-6 fatty acid; MT02, DNA replication inhibiting antibiotic; spermine NONOate, nitric oxide donor; TTO, tea tree oil; MRSA16791, methicillin-resistant chicken isolate; ATCC 29213, methicillin-sensitive strain; *rsp*, transcriptional regulator gene; BUSA2288, human nasal isolate; *saeS*, histidine kinase sensor of SaePQRS system gene.

b Values represent numbers of sRNAs with expression increased or decreased >3-fold (total = 303).

10.1128/mSphere.00439-20.5 DATA SET S2 Fold change and expression values for 303 sRNAs in 64 pairwise analyses. Download Data Set S2, XLSX file, 0.4 MB. Copyright © 2020 Sorensen et al. 2020 Sorensen et al. This content is distributed under the terms of the Creative Commons Attribution 4.0 International license.

To obtain a broad overview of the data generated, we first examined every pairwise comparison for each of the 303 sRNAs to identify conditions in which a >3-fold change in expression was observed (highlighted in yellow in Data Set S2). Table 2 lists how many of the 303 sRNAs showed >3-fold changes in expression in each of the 64 pairwise comparisons. The largest numbers of changes (>100 of 303 sRNAs) were observed when S. aureus cells growing as a biofilm were treated with the antibiotic fosfomycin. The large degree of variation in sRNA expression is similar to the global alteration in expression observed for all S. aureus genes upon fosfomycin treatment (7, 8). We next examined each of the 303 sRNAs to identify in how many of the 64 experiments a >3-fold expression change was observed for each of them. This analysis identified 11 sRNAs that showed >3-fold alterations in gene expression in 20 or more of the 64 pairwise analyses, suggesting that these are highly variably expressed sRNAs (Table 3). To our knowledge, none of these 11 variably expressed sRNAs have been studied in detail in S. aureus.

**TABLE 3:** sRNAs displaying altered expression in >20 of the 64 pairwise analyses

Feature ID	sRNA name	Chromosome region start position	Chromosome region end position	Total no. of sRNAs
SAUSA300s182	Sau-6079	461303	462824	26
SAUSA300s195	Sau-6745	2713518	2714477	25
SAUSA300s296	tsr32	2337921	2338072	25
SAUSA300s080	rsaOV	2696303	2696430	23
SAUSA300s078	rsaOT	2608637	2608828	22
SAUSA300s303	tsr39	2811277	2811330	22
SAUSA300s248	sRNA330	2257272	2257416	21
SAUSA300s289	tsr25	1442861	1443042	21
SAUSA300s168	Sau-76	643211	643332	20
SAUSA300s270	tsr6	120784	120897	20
SAUSA300s298	tsr34	2591031	2591131	20

Since the vast majority of the 303 annotated sRNAs have unknown function, we posit that meta-analysis of the data generated (Data Set S2) would be less meaningful than individual analysis of the results generated for an sRNA of interest. To demonstrate this principle, we investigated the data for the sRNA Teg41. Recent work by our group demonstrated a role for Teg41 in S. aureus virulence by which it influenced production of the alpha phenol-soluble modulins (αPSMs) (4). Although we demonstrated a role for Teg41 in αPSM production, expression of Teg41 itself was not examined. To gain some insight into the conditions that might influence Teg41 expression, we examined the data generated for Teg41 expression in our global sRNA expression analysis (Data Set S2). Teg41 expression was altered >3-fold in 13 of the 64 pairwise analyses (Table 4). The greatest change in expression (25.71-fold) was identified in comparing the results seen with S. aureus grown anaerobically at 0 and 240 min. These data suggest that Teg41 expression may be increased during anaerobic growth or might have a role in metabolism. Expression of Teg41 also increased 13.65-fold when the endoribonuclease RNaseY was deleted, suggesting that RNaseY may degrade Teg41 RNA when present. These results demonstrate the utility of the data presented here for gaining an insight into the conditions under which expression of a specific sRNA might be influenced.

**TABLE 4:** Conditions under which Teg41 expression is altered

Expt	Conditions	Fold change	Expression value 1^*a*^	Expression value 2^*a*^
001.6	UAMS-1 anaerobic 240 min vs *nos* KO 240 min	3.7	0.47	1.74
001.12	UAMS-1 anaerobic 0 min vs UAMS-1 anaerobic 120 min	3.86	77.38	299.04
001.13	UAMS-1 anaerobic 0 min vs UAMS-1 anaerobic 240 min	25.71	6.82	175.42
002.3	UAMS-1 ES vs UAMS-1 LE	5.5	299.73	1,648.35
003.1	Newman vs sulfide treated	4.51	566.14	125.58
003.3	Newman vs nitroxyl treated	3.02	371.41	123.06
003.4	Newman vs calprotectin treated	3.61	211.92	765.35
005.4	JE2 vs patient S nasal	3.07	19.53	6.36
007.2	HG003 vs *rsaE* plasmid complementation uninduced	3.57	256.33	916.27
007.4	*rsaE* mutant vs *rsaE* plasmid complementation uninduced	3.24	255.62	828.92
014.1	IPLA 1 biofilm vs treated with phage phiIPLA-RODI	4.94	48.48	9.81
017.1	PR01 vs RNase Y deletion	13.65	73.12	997.88
018.1	MRSA BD02-25 vs delta *rsp*	8.75	9.95	87.05

a Expression values are expressed in RPKM. Expression value 1 and expression value 2 represent the expression values of Teg41 under the two conditions tested. For details of each specific condition, see Supplemental Data Set S2.

### Global analysis of sRNA stability and degradation.

**FIG 2:**
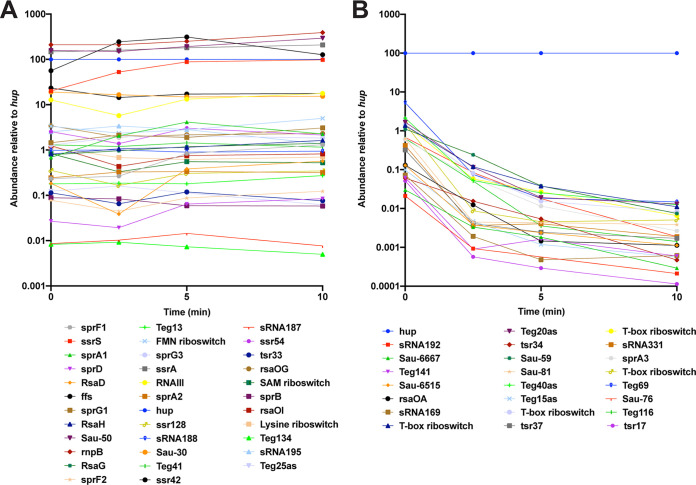
Analysis of sRNA stability. All sRNA expression values were normalized using the expression values for *hup*, which is included as a comparison (blue line). (A) Highly stable sRNAs. Thirty four sRNAs were deemed highly stable based on an overall stability profile similar to that of *hup*. The highly stable sRNAs were either more stable than *hup* or their stability was 2-fold less than that of *hup*. FMN, flavin mononucleotide; SAM, *S*-adenosylmethionine. (B) Highly unstable sRNAs. A total of 23 sRNAs were deemed highly unstable, with stability 100-fold less than that of *hup*.

RNA stability and degradation are components of an underappreciated method of genetic regulation whereby the rate of RNA turnover can be altered in response to genetic and environmental factors (9, 10). While degradation of mRNA transcripts is being increasingly studied, the stability and degradation of sRNA molecules are less so. One of the RNA-Seq studies used in the sRNA expression analysis described above (11) included a series of samples where wild-type S. aureus was treated with rifampin to arrest transcription and RNA was isolated both before the addition of rifampin (i.e., *t* = 0) and at 2.5, 5, and 10 min after rifampin treatment. These data sets were originally used to examine the degradation and stability of mRNA transcripts; however, sRNAs were not examined. We reanalyzed these data sets, comparing the levels of stability of the 303 sRNAs by normalizing the total RNA abundances over time to that of the housekeeping gene *hup*, as outlined in the original study (11). We then determined the relative stability of each sRNA by taking the abundance value after 10 min (*t*_10_) and dividing by the initial value measured at *t*_0_ (Data Set S3). Transcripts with a value greater than 0.5 were considered to be highly stable as the RNA degradation value determined for these transcripts represents no less than 50% (2-fold) of that observed for the *hup* transcript over the course of the experiment (Fig. 2A). Conversely, sRNAs with *t*_10_/*t*_0_ values of <0.01 (RNA decay, >99%) were considered to represent highly unstable RNA and were rapidly degraded (Fig. 2B). We acknowledge that the designations “highly stable” (Fig. 2A) and “highly unstable” (Fig. 2B) are somewhat arbitrary and reflective only of the conditions under which this experiment was performed (strain SA564, mid-exponential phase, Mueller-Hinton media) (11). Nonetheless, this analysis provides a valuable snapshot of the level of sRNA stability seen under the conditions tested.

10.1128/mSphere.00439-20.6 DATA SET S3 Relative stabilities of 303 sRNAs following rifampicin treatment. Download Data Set S3, XLSX file, 0.1 MB. Copyright © 2020 Sorensen et al. 2020 Sorensen et al. This content is distributed under the terms of the Creative Commons Attribution 4.0 International license.

**FIG 3:**
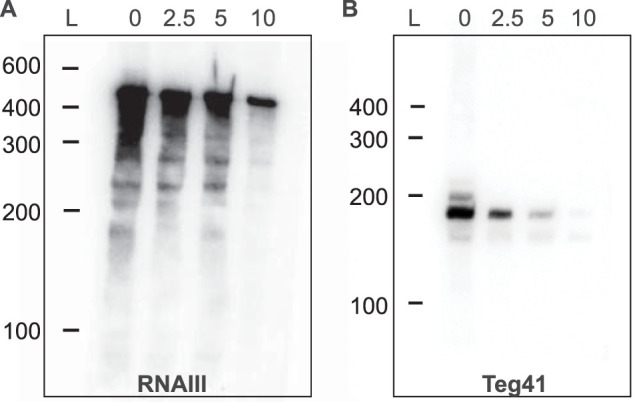
Northern blot analysis of selected stable sRNAs. (A) RNAIII. (B) Teg41. Order of lanes: L (RNA ladder), 0 (0 min sample—prior to rifampin addition), 2.5 (2.5 min after rifampin addition), 5 (5 min after rifampin addition), 10 (10 min after rifampin addition). The T_10_/T_0_ values for RNAIII and Teg41 were 1.39 and 0.89, respectively.

A total of 34 sRNAs were found to be highly stable after employing our cutoffs (Fig. 2A). Interestingly, many of the stable sRNAs are well characterized and have known functions, such as ssrA (transfer-messenger RNA [tmRNA]), which functions in protein production; the signal recognition particle component ffs (4.5S RNA); and ssrS (6S RNA), which is a global transcriptional regulator. These results agree with work previously reported by Roberts et al. and performed in the UAMS-1 background, where tmRNA was shown to be highly stable (9). The results of the study reported by Roberts et al. also demonstrated that *rnbP*, the RNA component of RNase P, is highly stable, which was also observed in our analysis (Fig. 2A). Additionally, our analysis identified a number of sRNAs involved in virulence regulation that were also highly stable such as RNAIII, Teg41, and ssr42. To experimentally validate our results for stable sRNAs, we performed Northern blotting over time on the RNAIII and Teg41 transcripts, after treatment with rifampin. Although our experimental analysis was performed in a different S. aureus background (i.e., USA300 AH1263), the results confirm that each of these transcripts was stable. The majority of S. aureus RNAs have a half-life of <2.5 min (10); however, a band was detected for RNAIII even after 10 min whereas Teg41 was found to be present through the 5-min time period and was barely detected at 10 min (Fig. 3). Twenty-three sRNAs were deemed to be “highly unstable” by our analysis (Fig. 2B). Most (if not all) of these unstable sRNAs have no known role in S. aureus.

This analysis, while limited in terms of strains and growth conditions, provides valuable insight into the stability of sRNAs in S. aureus. The results confirm the stability of RNAs that had previously been shown to be stable, validating the analytical approach. In addition, a large number of sRNAs with unknown function were shown to be highly stable. Additional investigation will be required to determine their biological activity, as their degree of stability suggests that they may play important roles.

### Identification of sRNAs that potentially encode proteins/peptides.

It is not uncommon for sRNAs to have dual functions in the bacterial cell. RNA can exert a regulatory function; in addition, a small open reading frame(s) (ORF[s]) found within the sRNA can encode functional peptides. Perhaps the best example of such a dual-function sRNA in S. aureus is RNAIII (which also encodes the delta toxin). We hypothesize that additional sRNAs, included in our original sRNA annotation study (2), could represent dual-function sRNAs that also encode peptides. In addition, it is possible that some of the sRNA annotations added in our original study represent protein coding genes that were omitted from the original genome annotation but added in our study as sRNAs. To investigate this further, and due to the availability of the data, we reanalyzed the results of a ribosome profiling (Ribo-seq) study performed previously by Basu and Yap (12). Our goal was to determine the degree of ribosome binding to the 303 sRNAs in the USA300 genome. Ribosome profiling identifies mRNA transcripts that are actively being translated (and therefore are protected by a ribosome) under the conditions tested. We reasoned that any sRNAs identified at a high frequency in the Ribo-seq data set might have the potential to code for small peptides or proteins.

**FIG 4:**
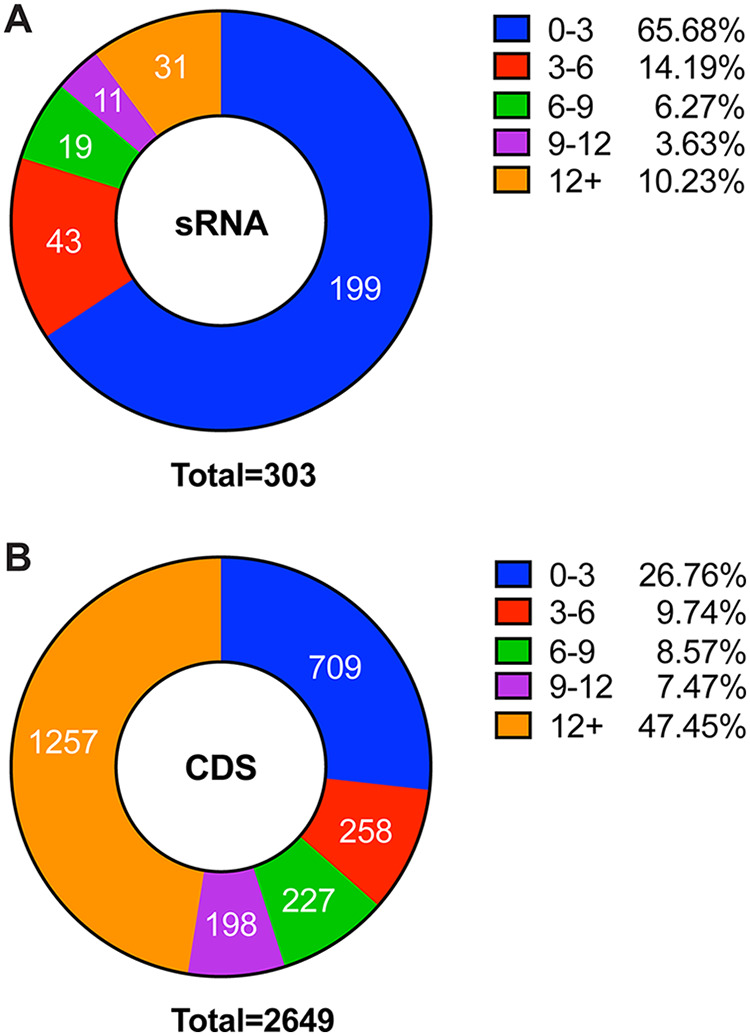
Analysis of sRNA ribosome protected fragments. (A) RPF/expression ratios of 303 sRNAs from S. aureus. A total of 65.68% of sRNAs had an RPF/expression ratio of >3. (B) RPF/expression ratios of 2,649 CDS genes from S. aureus.

The Ribo-seq data were reanalyzed by mapping the ribosome protected fragments (RPFs) to the S. aureus genome (containing sRNA annotations) and calculating RPKM (reads per kilobase per million) values for each sRNA. Next, RNA-Seq transcriptomic data sets, corresponding to the Ribo-seq data sets, were analyzed and expression values were calculated for each sRNA. RPKM values from the Ribo-seq experiment were divided by expression values from the RNA-Seq analysis, resulting in an RPF/expression ratio (Data Set S4). Normalizing the Ribo-seq data in this way accounts for variations in the expression level of each sRNA (12). The majority of sRNAs analyzed (199 of the 303 or 65.68%) had an RPF/expression ratio value between 0 and 3 (Fig. 4A), indicating that they likely do not encode peptides and are true sRNAs. A total of 43 sRNAs (14.19%) had a ratio between 3 and 6, 19 (6.27%) had a ratio between 6 and 9, 11 (3.63%) had a ratio between 9 and 12, and 31 (10.23%) had a ratio greater than 12 (Fig. 4A). To investigate if this distribution is indicative of noncoding RNAs, we repeated the analysis and examined the RPF/expression ratio of all coding DNA sequences (CDS) on the S. aureus genome. Results show a much lower percentage of CDS genes with an RPF/expression ratio between 0 and 3 (26.76% compared to 65.68% for sRNA genes; Fig. 4B). Conversely, the percentage of CDS genes with an RPF/expression ratio greater than 12 was 47.45%, compared with 10.23% for sRNA genes. On the basis of these results, we consider it probable that RNAs with a RPF/expression ratio between 0 and 3 have few to no ribosomes bound and likely represent noncoding sRNAs. sRNAs with a higher RPF/expression ratio may potentially encode proteins or peptides.

10.1128/mSphere.00439-20.7 DATA SET S4 RPF/expression ratio for 303 sRNAs. Download Data Set S4, XLSX file, 0.1 MB. Copyright © 2020 Sorensen et al. 2020 Sorensen et al. This content is distributed under the terms of the Creative Commons Attribution 4.0 International license.

**FIG 5:**
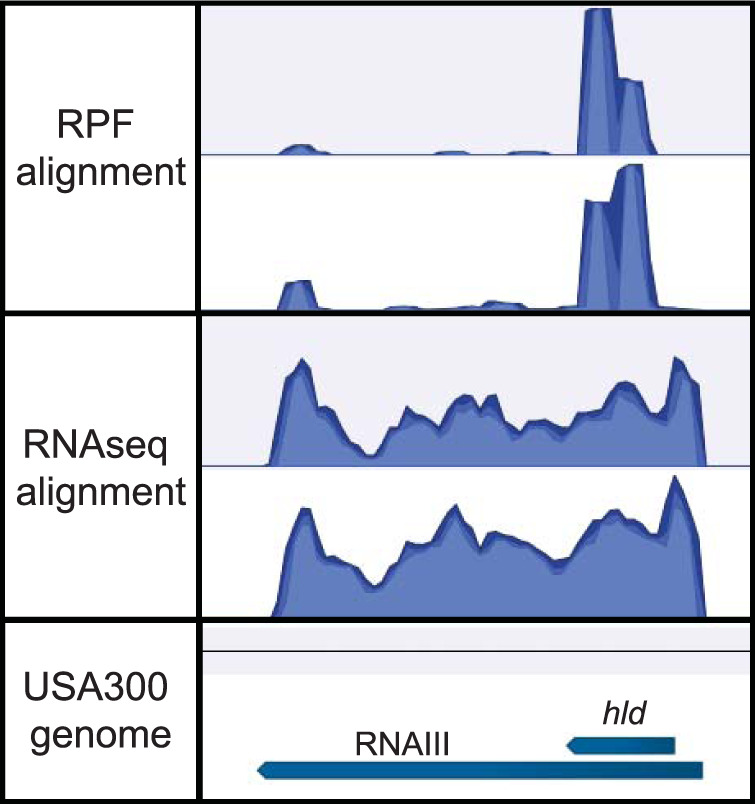
Ribosome profiling data for the RNAIII locus. Visualization of reads mapped to the RNAIII locus is provided. RPF alignment, read alignment of duplicate ribosome protected fragment data sets; RNAseq alignment, read alignment of duplicate RNA-Seq transcriptomic data sets. Mapping was generated using the CLC Genomics Workbench software package.

To further investigate the validity of the approach outlined above, we examined the RPF/expression data for RNAIII (which is known to encode a peptide) and also examined RPF/RNA-Seq read alignment data for the RNAIII locus in S. aureus. The RPF/expression ratio for RNAIII was 4.33, which was slightly lower than expected. An examination of the RPF/RNA-Seq read alignment data for the RNAIII locus shows a clear increase in the number of reads mapping to the *hld* locus in the RPF data set compared to the RNA-Seq data set (Fig. 5). The lower-than-expected RPF/expression ratio observed for RNAIII might have resulted from the fact that the *hld* coding sequence comprises a small portion of the entire RNA molecule (135 of 551 nucleotides, 24.5%). It also implies that any RNA with an RPF/expression ratio of >3 may potentially encode a protein/peptide.

Taken together, the results from our reanalysis of the ribosome profiling data set indicate that the absolute RPF/expression ratio does not automatically pinpoint translated and untranslated RNAs; however, they do indicate that a RPF/expression ratio value below 3 is suggestive of noncoding transcripts whereas values above 3 may indicate the potential to encode proteins.

### Confirmation that specific *tsr* genes encode peptides.

Previous work from our group identified 39 novel sRNAs (named *tsr1* to *tsr39*) in S. aureus strain USA300 FPR (2). In annotating these sRNAs on the S. aureus genome, we noted that the chromosomal loci corresponding to *tsr9*, *tsr17*, *tsr18*, *tsr21*, *tsr22*, *tsr35*, and *tsr37* were annotated as protein coding genes in S. aureus strains MRSA252 and NCTC 8325 (2). Consequently, it is possible that these genes encode proteins in addition to/instead of being sRNAs. In examining the ribosome profiling data, we noticed that a large number of *tsr* sRNAs had an RPF/expression ratio above 3, including six of the seven *tsrs* listed above. On the basis of this result, we hypothesized that *tsr9*, *tsr17*, *tsr18*, *tsr21*, *tsr22*, *tsr35*, and *tsr37* encode proteins/peptides.

To experimentally investigate this hypothesis, we first examined the sequence of each *tsr* for potential open reading frames (ORFs). ORFs identical to the genes annotated at the corresponding loci in MRSA252 and NCTC 8325 were identified in each of the *trs* genes. We next cloned each *tsr* gene along with its native promoter into the high-copy-number pMK4 plasmid and tagged the predicted ORF on the C terminus with a 6× histidine tag. The *tsr21* locus contained two unannotated potential ORFs (in addition to one annotated ORF); therefore, we individually tagged the previously unannotated ORFs and named them *tsr21B* and *tsr21C*, bringing the total number of tagged potential *tsr* peptides to eight.

**FIG 6:**
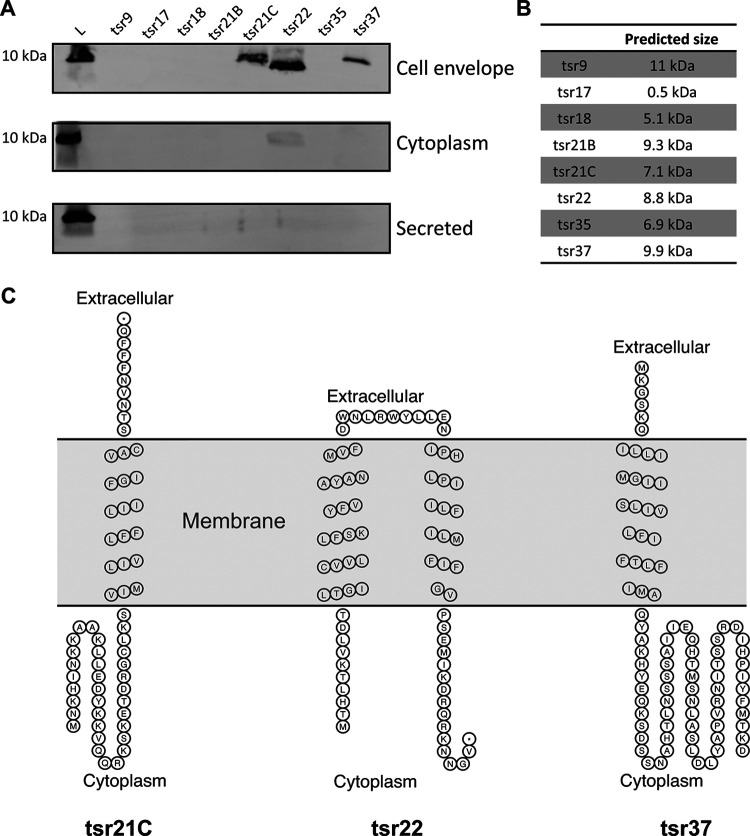
Western immunoblot detection of *tsr*-encoded potential small peptides. (A) Western blot analysis, performed using an anti-his antibody, to detect His-tagged, *tsr*-encoded peptides/proteins. Protein samples from the cell envelope, cytoplasm, and secreted fraction were analyzed. (B) Table of predicted sizes of potential *tsr*-encoded small peptides. (C) Predicted membrane topology of peptides encoded by *tsr21C*, *tsr22*, and *trs37*. All three peptides are predicted to contain transmembrane regions. Membrane topology was predicted using SACS MEMSAT2 Transmembrane Prediction (18).

The overexpression plasmids were transformed into wild-type S. aureus (USA300 AH1263) and the resulting strains grown for 4 h. Cultures were centrifuged to collect bacterial cells, and protein samples were prepared from the cytoplasmic, cell envelope, and secreted fractions. Western blot analysis was performed using an antihistidine antibody to identify putative small proteins. Bands corresponding in size to the predicted peptides encoded by *tsr21C*, *tsr22*, and *tsr37* were identified in the cell envelope fraction (Fig. 6A and B). No peptides were detected for *tsr9*, *tsr17*, *tsr18*, *tsr21B*, or *tsr35* in any of the protein fractions (for *tsr17*, we acknowledge that it is unlikely that a peptide of 0.5 kDa would be detected on this gel). An analysis of the predicted amino acid sequence of the *tsr21C*, *tsr22*, and *tsr37* peptides shows that all three peptides have predicted transmembrane helices (Fig. 6C). Collectively, these data suggest that the genes coding for *tsr21C*, *tsr22*, and *tsr37* may actually code for small peptides that are located in the bacterial cell membrane. The Western blot analysis was performed under only one set of growth conditions (i.e., in the exponential phase in tryptic soy broth [TSB]), and so the failure to detect *tsr9*, *tsr17*, *tsr18*, *tsr21B*, or *tsr35* in any of the protein fractions may simply indicate that these proteins were not expressed under the conditions tested.

**FIG 7:**
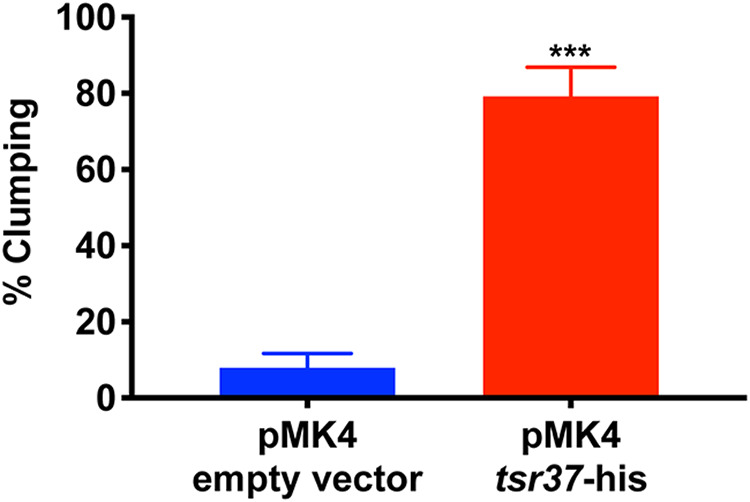
Quantitative analysis of clumping phenotype observed in *tsr37* overexpressor strain. A clumping assay was performed using S. aureus strain AH1263 overexpressing *tsr37* (pMK4 *tsr37*-his), and results were compared to those seen with an empty vector control (pMK4 empty vector). The proportion of clumping of the empty vector control after 2 h was 7.9% compared to 79.2% for the *tsr37*-overexpressing strain.

Interestingly, in growing the strains outlined above for the Western blot analysis, we observed that S. aureus cultures overexpressing histidine-tagged *tsr37* rapidly autoaggregated and settled to the bottom of the culture tube. For simplicity, we refer to this process as “clumping,” but note that no agglutinating agent (such as fibrinogen or plasma) had been added to these cultures. To quantify the results, an aggregation assay (described previously [13]) was performed to compare the degree of clumping seen with the *tsr37* overexpressing strain to that seen with a pMK4 empty vector control. Exponentially growing cells were washed and resuspended in 1 ml of phosphate-buffered saline (PBS), and an initial measurement of the optical density at 600 nm (OD_600_) of the sample was performed. After 2 h, 100 μl was withdrawn from the top of each sample and the OD_600_ measurement performed again. Percent clumping of each strain was then determined. A 10-fold increase in clumping was observed in the strain overexpressing the His-tagged *tsr37* peptide (79.2% clumping at 2 h) compared to empty vector controls (7.9% clumping at 2 h) (Fig. 7). These data suggest (i) that *tsr37* encodes a novel small peptide and (ii) that *tsr37* has a biologically meaningful role in the cell. While it is tempting to speculate that the clumping phenotype observed upon *tsr37* overexpression was due to the activity of the *tsr37*-encoded peptide, it remains possible that *tsr37* functions exclusively as an RNA molecule and that the clumping phenotype is mediated via the *tsr37* sRNA. Work is ongoing in our lab to investigate the nature and biological activity of *tsr37*.

### Analysis of *in vivo* RNA-Seq data sets.

**FIG 8:**
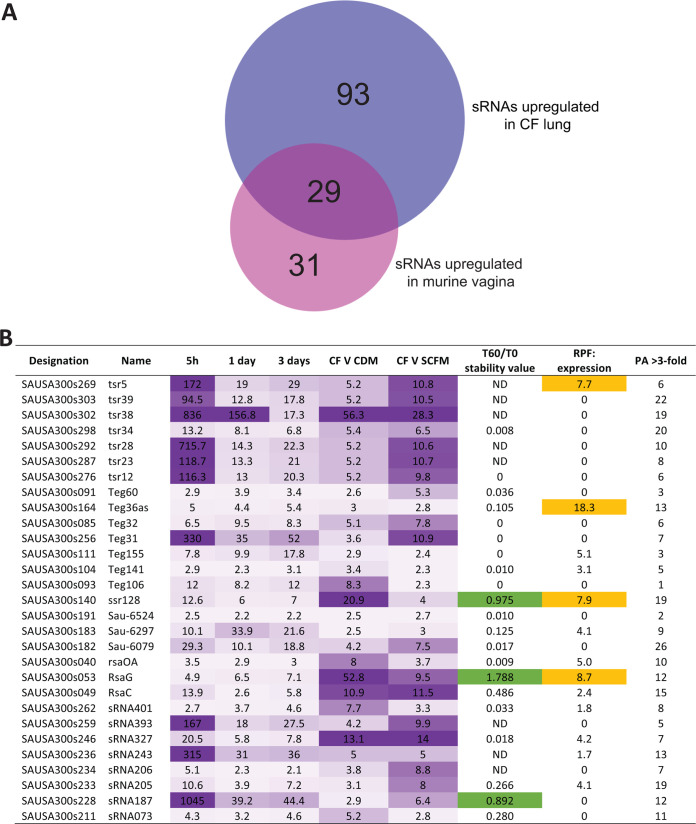
Analysis of sRNA expression *in vivo*. (A) A total of 93 sRNAs were upregulated >2-fold in the CF lung, whereas 60 sRNAs were upregulated >2-fold in the mouse vagina. A total of 29 sRNAs were upregulated in both data sets. (B) Table of 29 sRNAs upregulated in both *in vivo* data sets. Fold increase in expression in the vagina is indicated for each time point and compared to CDM/SCFM data. Also included are stability values (taken from Data Set S3), RPF/expression ratios (taken from Data Set S4), and the number of pairwise analyses where a >2-fold change in expression was observed (taken from Data Set S2).

Recent advances in RNA-Seq technology, including reductions in cost, the ability to generate more reads, and the ability to generate data from small amounts of input RNA, have led to the application of this method *in vivo* to perform transcriptomic analysis of bacteria during the infectious process. Recently, two studies have utilized RNA-Seq to examine the transcriptional response of S. aureus *in vivo*. Work by Ibberson and Whiteley examined the S. aureus transcriptome in the lung of patients with cystic fibrosis (CF) (14), while Deng et al. examined the S. aureus transcriptome during vaginal colonization (15). The RNA-Seq data sets for these two studies were not yet available at the time that we performed the original search of the GEO database (for our global sRNA expression analysis described above). We hypothesized that a reanalysis of these data sets could potentially identify sRNAs with altered expression *in vivo* (and, consequently, sRNAs that may play a role *in vivo*); therefore, we analyzed both data sets with our updated reference genome file. For analysis of the CF data set, we compared the transcriptomes of the nine samples isolated from CF patients with transcriptomes from nine S. aureus cultures grown in chemically defined media (CDM) and from nine S. aureus cultures grown in synthetic cystic fibrosis media (SCFM). We examined the data for sRNAs that were upregulated a minimum of 2-fold in the CF lung compared to both CDM and SCFM and found that a total of 122 sRNAs met our cutoffs (Fig. 8; see also Data Set S5). Similarly, for the vaginal persistence data set we compared S. aureus growing in laboratory media to samples isolated from the vagina of mice at 5 h, 1 day, and 3 days postinoculation. We utilized multiple comparisons to determine which sRNAs were upregulated a minimum of 2-fold at each of the three time points in the vagina. These cutoffs led to the identification of 60 sRNAs that were consistently upregulated during vaginal persistence (Fig. 8; see also Data Set S6). Finally, we cross-referenced these analyses to identify sRNAs that were upregulated in both studies (and that therefore may be broadly important for S. aureus *in vivo*). A total of 29 sRNAs were identified as upregulated in both the CF lung and the vagina compared to the corresponding *in vitro* conditions (Fig. 8B). These sRNAs are excellent candidates for future studies as they may play important roles during *in vivo* colonization and/or virulence. Interestingly, very few (if any) of the 29 sRNAs identified have been studied in detail or have any known role in S. aureus. With that in mind, we compiled all of the data generated in this study for these 29 *in vivo*-expressed sRNAs, including their relative stabilities, RPF/expression ratios, and numbers of pairwise analyses where they demonstrated >3-fold variation in expression (Fig. 8B). Of particular interest, we observed that both ssr128 and RsaG were highly stable and had high RPF/expression ratios (7.9 and 8.7, respectively), suggesting the potential for these sRNAs to encode proteins. Indeed, when we examined the DNA sequence of ssr128 and RsaG, we observed ORFs that could potentially encode proteins of 56 and 25 amino acids, respectively. While not conclusive, these data suggest that ssr128 and RsaG warrant further investigation and that each could function as an sRNA and/or protein coding transcript.

10.1128/mSphere.00439-20.8 DATA SET S5 Expression values of 303 sRNAs in CF lung isolates. Download Data Set S5, XLSX file, 0.2 MB. Copyright © 2020 Sorensen et al. 2020 Sorensen et al. This content is distributed under the terms of the Creative Commons Attribution 4.0 International license.

10.1128/mSphere.00439-20.9 DATA SET S6 Expression values of 303 sRNAs in vaginal colonization model. Download Data Set S6, XLSX file, 0.1 MB. Copyright © 2020 Sorensen et al. 2020 Sorensen et al. This content is distributed under the terms of the Creative Commons Attribution 4.0 International license.

### Conclusions and limitations.

The variety of ways in which RNA-Seq can be used is rapidly expanding, and the versatility of the technique is reflected in the variety of analyses presented here. We examined variation in expression of sRNAs under different environmental/growth conditions and in different genetic backgrounds and then went on to analyze sRNA stability/degradation and binding of ribosomes. Finally, we reexamined two recently published studies that used RNA-Seq to examine bacterial gene expression *in vivo* and identified sRNAs that are upregulated in each niche. The results generated represent a useful guide for future studies, but we also acknowledge the limitations regarding their interpretation. In certain cases, the experimental data analyzed arose from a single set of growth conditions (e.g., the RNA stability and Ribo-seq data sets). The stabilities of sRNAs and their potentials to bind ribosomes and to be translated might, and most likely do, differ under differing growth conditions (9–11). In addition, while the USA300 FPR3757 genome file was used as a reference throughout, the RNA-Seq data sets used in this study were not all generated in the same strain of S. aureus. We acknowledge the limitation that this represents; however, comparative analyses were performed only on data sets from the same study (and thus the same strain); therefore, any error introduced (due to differences in the experimental strain and reference genome) should be consistent throughout.

## MATERIALS AND METHODS

### Bacterial strains and plasmids.

The bacterial strains and plasmids used in this study are listed in Table 5. S. aureus and E. coli strains were grown routinely at 37°C with shaking in tryptic soy broth (TSB) and lysogeny broth (LB), respectively. When necessary, antibiotics were added at the following concentrations: chloramphenicol at 10 μg/ml and ampicillin at 100 μg/ml.

**TABLE 5:** Strains and plasmids

Strain or plasmid	Characteristic(s)	Reference or source
Strains		
*S. aureus* AH1263	USA300 LAC isolate cured of plasmid LAC-p03	35
*S. aureus* RN4220	Restriction-deficient transformation recipient	36
*S. aureus* RKC885	AH1263 pTsr17-his	This study
*S. aureus* RKC886	AH1263 pTsr18-his	This study
*S. aureus* RKC887	AH1263 pTsr21B-his	This study
*S. aureus* RKC888	AH1263 pTsr21C-his	This study
*S. aureus* RKC889	AH1263 pTsr22-his	This study
*S. aureus* RKC681	AH1263 pTsr37-his	This study
*S. aureus* RKC895	AH1263 pTsr9-his	This study
*E. coli* DH5α	Cloning strain	Invitrogen

Plasmids		
pMK4	Gram-positive shuttle vector (CM^r^)^*a*^	37
pRKC679	pMK4_Tsr37_6x-his (vector overexpressing His-tagged *tsr37* from its native promoter)	This study
pRKC885	pMK4_Tsr17_6x-his (vector overexpressing His-tagged *tsr17* from its native promoter)	This study
pRKC886	pMK4_Tsr18_6x-his (vector overexpressing His-tagged *tsr18* from its native promoter)	This study
pRKC887	pMK4_Tsr21B_6x-his (vector overexpressing His-tagged *tsr21B* from its native promoter)	This study
pRKC888	pMK4_Tsr21C_6x-his (vector overexpressing His-tagged *tsr21C* from its native promoter)	This study
pRKC889	pMK4_Tsr22_6x-his (vector overexpressing His-tagged *tsr22* from its native promoter)	This study
pRKC892	pMK4_Tsr9_6x-his (vector overexpressing His-tagged *tsr9* from its native promoter)	This study

a CM^r^, chloramphenicol resistance.

### Bioinformatics and sRNA expression analysis.

All data sets were downloaded directly from GEO. RNA-Seq data analysis was performed using the CLC Genomics Workbench software platform (Qiagen). The genome file containing sRNA annotations for S. aureus USA300 was previously published by our group and is available online (2). RNA-Seq data analysis was carried out using a protocol previously published by our research group (16). The resulting data were exported, and only the data for sRNAs were further analyzed.

### RNA stability analysis and normalization.

To evaluate RNA stability, data were downloaded from GEO (accession number SRP058249) (11). Results from the RNA-Seq analysis were used for a multigroup, unpaired comparison to generate expression values in RPKM. Mean expression values were determined for each sRNA at times 0 min, 2.5 min, 5 min, and 10 min after rifampin treatment. The mean expression value for each sRNA was then normalized to the expression value of *hup* at the same time point in order to account for global RNA degradation over time. After normalization, stable transcripts were determined by dividing the expression value at time point 10 min (*t*_10_) by the expression value of the same sRNA at time point 0 min (*t*_0_) to generate a *t*_10_/*t*_0_ value. Selected stable sRNAs (34 in total) had a *t*_10_/*t*_0_ value greater than 0.5, and the selected unstable sRNAs (23 in total) had a *t*_10_/*t*_0_ value of less than 0.01.

### RNA stability and Northern blotting.

Cultures of USA300 strain AH1263 were grown in tryptic soy broth (TSB) for 2.5 h. At time point 0, 3 ml of culture was removed and placed into two 1.5-ml tubes and the cells were pelleted by centrifugation at 15,000 × *g* for 1 min. The supernatant was immediately aspirated, and the pellet was flash frozen using liquid nitrogen. Also at time zero, rifampin (final concentration of 200 μg/ml) was added to the culture. The sample preparation process was repeated at time points 2.5, 5, and 10 min. Pellets were stored at −80°C for subsequent RNA isolation. RNA was isolated as previously described (16), and samples were stored at −80°C.

Northern blotting to monitor the stability of selected sRNAs over time was performed as described previously (2). Briefly, 4 μg of total RNA obtained from the RNA stability assay was loaded in each lane of a 10% polyacrylamide gel (7 M urea, 1× Tris-borate-EDTA [TBE]) and separated by gel electrophoresis. The samples were then transferred via electroblotting to an Immobilon NY+ nylon membrane (Millipore) and cross-linked to the membrane by the use of UV irradiation. Radiolabeled probes for Teg41 and RNAIII were generated as previously described (4). Teg41 was amplified by PCR using primers 301 and 302, and the RNAIII transcript was amplified using primers 9 and 10. Products were labeled with α-^32^P using a random primed DNA labeling kit (Roche) and purified with illustra MicroSpin G-25 columns (GE Healthcare). Membranes were prehybridized (1 h, 45°C) in ULTRAhyb-Oligo buffer (Ambion) and then incubated with radiolabeled probe overnight. After incubation, membranes were washed with 2×, 1×, and 0.5× SSC buffer (1× SSC is 0.15 M NaCl plus 0.015 M sodium citrate) at 45°C for 15 min each time and visualized using a phosphorimager screen.

### Analysis of ribosome protected fragments.

Ribosome profiling (Ribo-seq) data were downloaded from GEO (accession number SRP065033). Both the Ribo-seq data and the accompanying RNA-Seq data were analyzed as described previously (16). The expression values were normalized by dividing the Ribo-seq expression value (in RPKM) by the corresponding RNA-Seq expression value to generate an RPF/expression ratio. This normalization accounts for the relative abundance of each RNA transcript. These RPF/expression ratio values were then evaluated and used to identify potential small peptides.

### Western blotting.

For Western blotting, bacterial cultures were grown to mid-exponential phase (3 h). Cells were pelleted and the supernatant was subjected to trichloroacetic acid (TCA) precipitation as described previously (17). Bacterial pellets were resuspended in 1 ml PBS and treated with 20 μl of lysostaphin for 30 min at 37°C. Lysates were then treated twice with 5 μl DNase I for 30 min at 37°C. Cellular debris was pelleted, and the supernatant (cytoplasmic fraction) was removed to a new tube. The pellet was washed with PBS and resuspended in 1 ml of 8 M urea. For TCA precipitation, 9 ml of supernatant was combined with 1 ml of trichloroacetic acid and incubated at 4°C for 24 h. Precipitated proteins were pelleted at 4°C and washed three times with ice-cold acetone. The protein pellet was resuspended in 500 μl of 8 M urea. Equal quantities of protein, as determined by a Bradford assay, were electrophoresed on a polyacrylamide gel. The gel was then transferred using semidry transfer at 10 V for 30 min onto a polyvinylidene fluoride membrane. The membrane was blocked for 1 h using phosphate-buffered saline supplemented with 1% Tween (PBST) and 10% milk, with rocking, at room temperature. The membrane was incubated for 16 h with rabbit anti-6×His antibody–PBST–10% milk, with rocking, at 4°C. The membrane was washed three times with PBST for 10 min each time. The membrane was incubated with 1:10,000 of a goat anti-rabbit fluorescent antibody for 1 h, with rocking, at room temperature. The membrane was washed three times with PBST and imaged using an Odyssey CLx imager (Li-Cor).

### Analysis of *in vivo* data sets.

*In vivo* RNA-Seq data sets were acquired from GEO under accession number SRP222773 for CF data and accession number SRP229518 for vaginal data (14, 15). For the CF lung data, sRNA expression values (in RPKM) from nine clinical isolate samples were averaged and compared to the averaged sRNA expression values from nine S. aureus cultures grown in chemically defined media (CDM) as well as to those from nine samples from synthetic cystic fibrosis media (SCFM). A 2-fold cutoff value was employed to identify sRNAs with increased expression in the CF lung compared to both media. For the vaginal data, results from S. aureus growing in laboratory media were compared to results from samples isolated from the vagina of mice at 5 h, 1 day, and 3 days postinoculation. A 2-fold cutoff value was used to identify sRNAs with consistently increased expression in the vagina.
