# Supplementary material for: Reading between the Lines: Utilizing RNA-Seq Data for Global Analysis of sRNAs in Staphylococcus aureus
Source: mSphere. 2020 Jul 29;5(4):e00439-20. doi: 10.1128/mSphere.00439-20 (PMC7392542; doi:10.1128/mSphere.00439-20)
Supplement: TEXT S1 [file mSphere.00439-20-s0001.docx]

**Supplemental materials and methods**

**Data acquisition**

To identify previously published RNA-seq data sets for use in this study the following searches were performed in the Gene Expression Omnibus (GEO) online data repository. On October 31, 2018 the search term “*Staphylococcus aureus* RNAseq” was entered into GEO, which generated 30 hits (Supplemental File Search Results 1). On October 31, 2018 the search term “*Staphylococcus aureus* RNA-seq” was used, which generated 36 hits (Supplemental File Search Results 2). Using these data sets as a starting point we selected data sets for further analysis based on the following criteria:

1. Samples were from *Staphylococcus aureus* genus
2. Samples were provided in at least 2 replicates
3. Samples were complete transcriptomic RNA-seq samples (e.g. not dRNA-seq)
4. Samples could be placed in comparable groups based upon condition or genotype (e.g., WT vs mutant, or WT in mid log vs WT in stationary phase)
5. No previous analysis of *S. aureus* sRNAs had been performed with the same data

17 of the 30 search results from the “Staphylococcus aureus RNAseq” search (Supplemental File Search Results 1 #14-30) and 10 of the 36 search results from the “Staphylococcus aureus RNAseq” search (Supplemental File Search Results 2 #27-36) were individual data sets that were already accounted for and were therefore excluded. Studies that met our criteria were included, and given a “study number”. Studies that did not meet our criteria were excluded. The reasons for each exclusion are provided in Supplemental File Search Results 1 and 2.

**Generation of pairwise comparisons**.

All selected datasets within a study were examined and pairwise comparisons identified consisting of either (i) wild type vs. mutant, (ii) wild type vs. patient isolate, (iii) untreated vs. treated, (iv) standard growth conditions vs. altered growth conditions, (v) two different timepoints/phases of growth, (vi) standard growth vs. exposure to bacteriophage, (vii) antibiotic sensitive vs. resistant, or (viii) mutant vs. complement strains. Pairwise comparisons were made only between datasets from the same study in order to eliminate confounding variables due to growth conditions, sample preparation techniques, etc. that varied between groups. All pairwise comparisons are described in Table 2.

**Cloning and histidine tagging of *tsr* peptides**

Each *tsr gene,* including its native promoter, was amplified from USA300 genomic DNA (see primer table below). In each case the reverse primer added a hexahistidine (6xhis) tag to the C-terminus of the predicted peptide. Addition of the histidine tag enabled detection of any encoded peptides using an anti-histidine antibody. The tsr-his fragments were cloned into the shuttle vector pMK4 and transformed into chemically competent *E. coli* DH5α. Resulting colonies were screened for the insert via colony PCR and the correct insert was confirmed via plasmid sequencing. Both screening and sequencing utilized M13 forward and reverse primers. Confirmed plasmids were electroporated into RN4220 and subsequently phage transduced into AH1263 utilizing φ 11. Presence of the plasmid was confirmed in both RN4220 and AH1263 via colony PCR.

| Name | Sequence | Target |
| --- | --- | --- |
| #669 | AAAACTGCAGAAAATTAATGCGATGATTTTTAGC | tsr37 |
| #670 | CGGATCCTTAATGATGATGATGATGATGATCTTTTGTCATGAAATAAATGGG | tsr37 |
| #886 | AAAACTGCAGAAGTTTATGATATATTTAGA | tsr9 |
| #887 | CGGGATCCTTAATGATGATGATGATGATGAATGCTGAAGAATAATTT | tsr9 |
| #888 | AAAACTGCAGCTGAAATCTTAACTAATATT | tsr17 |
| #889 | CGGGATCCTTAATGATGATGATGATGATGTACTAAGATGAGCGACAG | tsr17 |
| #890 | AAAACTGCAGTATGTTCACCTCAAAATCAT | tsr18 |
| #891 | CGGGATCCTTAATGATGATGATGATGATGATTTAGTGTATCTTGGAT | tsr18 |
| #892 | AAAACTGCAGTTTGAAATTGTTCAAAACTT | tsr21B |
| #893 | CGGGATCCTTAATGATGATGATGATGATGGAATACATAAATGAATAG | tsr21B |
| #894 | AAAACTGCAGTTTGAAATTGTTCAAAACTT | tsr21C |
| #895 | CGGGATCCTTAATGATGATGATGATGATGCTGGAAGAAAAAGTTTAC | tsr21C |
| #896 | AAAACTGCAGTTTTTAAAATTTGTTTTTAA | tsr22 |
| #897 | CGGGATCCTTAATGATGATGATGATGATGAACACCGTTATTTTTCCT | tsr22 |
