# Supplementary material for: Reading between the Lines: Utilizing RNA-Seq Data for Global Analysis of sRNAs in Staphylococcus aureus
Source: mSphere. 2020 Jul 29;5(4):e00439-20. doi: 10.1128/mSphere.00439-20 (PMC7392542; doi:10.1128/mSphere.00439-20)
Supplement: TEXT S2 [file mSphere.00439-20-s0002.docx]

Search term “*Staphylococcus aureus* RNAseq”

Date of search Oct 31 2018

1. RNASeq analysis of untreated versus treated

**Included as Study #004**

(Submitter supplied) We report differential regulated genes in three pathogenic bacterial strains after treatment with a new antimicrobial.

Organism: Staphylococcus aureus; Streptococcus pyogenes; Enterococcus faecium

Type: Expression profiling by high throughput sequencing

Platforms: GPL23589 GPL19006 GPL20656 18 Samples

FTP download: GEO (XLSX) ftp://ftp.ncbi.nlm.nih.gov/geo/series/GSE121nnn/GSE121555/

SRA Run Selector: https://www.ncbi.nlm.nih.gov/Traces/study/?acc=PRJNA497790

Series Accession: GSE121555 ID: 200121555

2. Remapping the SRA: Drosophila melanogaster RNA-Seq data from the Sequence Read Archive

**Excluded – Exclusion based on criteria #1**

(Submitter supplied) The sequence read archive (SRA) contains over 52 terabases or 482 billion reads from Drosophila melanogaster (as of June 2018). These data are massively underused by the community and include 14,423 RNA-Seq samples, that is roughly 7 times the size of modENCODE. Currently the major challenge is finding high quality datasets that are suitable for inclusion in new studies. To help the community overcome this hurdle, we re-processed all D. more...

Organism: Drosophila melanogaster

Type: Third-party reanalysis; Expression profiling by high throughput sequencing

18 related Platforms 14423 Samples

FTP download: GEO (BW, GTF, TSV, TXT) ftp://ftp.ncbi.nlm.nih.gov/geo/series/GSE117nnn/GSE117217/

Series Accession: GSE117217 ID: 200117217

3. Response of nor and nos contributes to Staphylococcus aureus virulence and metabolism.

**Included as Study #001**

(Submitter supplied) CC30 S. aureus were subjected to NO stress and Anaerobic growth and produced different transcriptomes

Organism: Staphylococcus aureus

Type: Expression profiling by high throughput sequencing

Platform: GPL19006 30 Samples

FTP download: GEO (TXT) ftp://ftp.ncbi.nlm.nih.gov/geo/series/GSE114nnn/GSE114147/

SRA Run Selector: https://www.ncbi.nlm.nih.gov/Traces/study/?acc=PRJNA464411

Series Accession: GSE114147 ID: 200114147

4. Staphylococcus aureus bona fide sRNAs in the model strain HG003

**Excluded – Exclusion based on criteria #4**

(Submitter supplied) Accurate annotation of regulatory RNAs is a complex task but nevertheless essential as sRNA molecular and functional studies ensue from it. Several formerly considered small RNAs (sRNA) are now known to be parts of UTR transcripts. In light of experimental data, we review hundreds of Staphylococcus aureus putative regulatory RNAs. We pinpoint those that are likely acting in trans and are not expressed from the opposite strand of a coding gene. more...

Organism: Staphylococcus aureus

Type: Non-coding RNA profiling by high throughput sequencing

Platform: GPL16057 1 Sample

FTP download: GEO (GFF) ftp://ftp.ncbi.nlm.nih.gov/geo/series/GSE104nnn/GSE104971/

SRA Run Selector: https://www.ncbi.nlm.nih.gov/Traces/study/?acc=PRJNA414251

Series Accession: GSE104971 ID: 200104971

5. Regulation of gene expression by S. aureus and GAS (S. pyogenes) in murine bone marrow-derived dendritic cells (BMDC)

**Excluded – Exclusion based on criteria #1**

(Submitter supplied) We report the differential regulation of Th17-inducing cytokine genes in Dendritic cells by GAS and S. aureus

Organism: Mus musculus

Type: Expression profiling by high throughput sequencing

Platform: GPL18635 9 Samples

FTP download: GEO (TSV) ftp://ftp.ncbi.nlm.nih.gov/geo/series/GSE96nnn/GSE96086/

SRA Run Selector: https://www.ncbi.nlm.nih.gov/Traces/study/?acc=PRJNA378782

Series Accession: GSE96086 ID: 200096086

6. The Staphylococcus aureus nitric oxide synthase (saNOS) modulates respiratory metabolism and cell physiology

**Excluded – Exclusion based on criteria #2**

(Submitter supplied) Nitric oxide synthase (NOS) enzymes produce nitric oxide (NO), a highly reactive free radical capable of interacting with multiple cellular targets. Although saNOS contributes to Staphylococcus aureus virulence, as well as protection against exogenous oxidative stress and antimicrobials, the current mechanism behind these phenotypes is unknown. Here we report a previously-undescribed role for saNOS in modulating S. more...

Organism: Staphylococcus aureus

Type: Expression profiling by high throughput sequencing

Platform: GPL19476 3 Samples

FTP download: GEO (XLS) ftp://ftp.ncbi.nlm.nih.gov/geo/series/GSE77nnn/GSE77400/

SRA Run Selector: https://www.ncbi.nlm.nih.gov/Traces/study/?acc=PRJNA310224

Series Accession: GSE77400 ID: 200077400

7. Changes in relative transcript amounts caused by hydrogen sulfide treatment, calprotectin treatment, and deletion of CstR in Staphylococcus Aureus

**Included as Study #003**

(Submitter supplied) The Staphylococcus aureus strain Newman uses the dithiol-containing repressor CstR to sense sulfide stress via reactive sulfur species (RSS), allowing transcription of a mitochondrial-like sulfide oxidation system, the core of which is genetically linked to methicillin resistance determinants in MRSA strains. The cytoplasm maintains an excess of reduced relative to oxidized low molecular weight (LMW) thiols that are protective against oxidative stress and transition metal (Zn, Cd and Cu) toxicity, buffering these ions to low “free” concentrations via formation of coordination complexes. more...

Organism: Staphylococcus aureus

Type: Expression profiling by high throughput sequencing

Platforms: GPL19006 GPL17452 18 Samples

FTP download: GEO (TXT) ftp://ftp.ncbi.nlm.nih.gov/geo/series/GSE99nnn/GSE99432/

SRA Run Selector: https://www.ncbi.nlm.nih.gov/Traces/study/?acc=PRJNA388446

Series Accession: GSE99432 ID: 200099432

8. The ω Subunit Governs RNA Polymerase Stability and Transcriptional Specificity in Staphylococcus aureus

**Excluded – Exclusion based on criteria #2**

(Submitter supplied) Staphylococcus aureus is a major human pathogen that causes infection in a wide variety of sites within the human body. Its ability to adapt to the human host, and to produce a successful infection, requires precise orchestration of gene expression. While DNA-dependent RNA polymerase (RNAP) is generally well characterized, the role of several small accessory subunits within the complex has yet to be fully explored. more...

Organism: Staphylococcus aureus

Type: Expression profiling by high throughput sequencing

Platform: GPL19476 2 Samples

FTP download: GEO (XLSX) ftp://ftp.ncbi.nlm.nih.gov/geo/series/GSE87nnn/GSE87033/

SRA Run Selector: https://www.ncbi.nlm.nih.gov/Traces/study/?acc=PRJNA343254

Series Accession: GSE87033 ID: 200087033

9. TSS-EMOTE, a refined protocol for a more complete and less biased global mapping of transcription start sites in bacterial pathogens.

**Excluded – Exclusion based on criteria #4**

(Submitter supplied) Background Bacteria rely on efficient gene regulatory mechanisms to switch between genetic programs when they are facing new environments. Although this regulation can occur at many different levels, one of the key steps is the initiation of transcription. Identification of the first nucleotide transcribed by the RNA polymerase is therefore essential to understand the underlying regulatory processes, since this provides insight on promoter strength and binding sites for transcriptional regulators, and additionally reveals the exact 5' untranslated region of the transcripts, which often contains elements that regulate translation. more...

Organism: Klebsiella aerogenes; Acinetobacter baumannii; Staphylococcus aureus; Staphylococcus epidermidis

Type: Expression profiling by high throughput sequencing; Other

4 related Platforms 30 Samples

FTP download: GEO (TSV) ftp://ftp.ncbi.nlm.nih.gov/geo/series/GSE85nnn/GSE85110/

SRA Run Selector: https://www.ncbi.nlm.nih.gov/Traces/study/?acc=PRJNA336217

Series Accession: GSE85110 ID: 200085110

10. Transcriptomic Analysis of Staphylococcal sRNAs: Insights into Species Specific Adaption and the Evolution of Pathogenesis

**Excluded – Exclusion based on criteria #5**

(Submitter supplied) Next-generation sequencing technologies have dramatically increased the rate at which new genomes are sequenced. Accordingly, automated-annotation programs have become adept at identifying and annotating protein coding regions, as well as common and conserved RNAs. Additionally, RNAseq techniques have advanced our ability to identify and annotate regulatory RNAs (sRNAs), which remain significantly understudied. more...

Organism: Staphylococcus aureus; Staphylococcus carnosus; Staphylococcus epidermidis

Type: Non-coding RNA profiling by high throughput sequencing

Platforms: GPL21408 GPL19476 GPL21407 3 Samples

FTP download: GEO (XLSX) ftp://ftp.ncbi.nlm.nih.gov/geo/series/GSE77nnn/GSE77567/

SRA Run Selector: https://www.ncbi.nlm.nih.gov/Traces/study/?acc=PRJNA311210

Series Accession: GSE77567 ID: 200077567

11. RNA-Seq-mediated transcriptome analysis of Staphylococcus aureus Newman wild-type, walKD119A, walKV149A and DHBP-treated wild-type strains

**Included as Study #006**

(Submitter supplied) We reported the RNA-Seq results of Staphylococcus aureus Newman wild-type, walKD119A, walKV149A and DHBP-treated wild-type strains. We found that mutations of the potential signal transduction residues of WalK attenuate the activity of walKR two-component system, whereas DHBP supplementation activates this two-component system.

Organism: Staphylococcus aureus subsp. aureus str. Newman

Type: Expression profiling by high throughput sequencing

Platform: GPL20326 8 Samples

FTP download: GEO (TXT) ftp://ftp.ncbi.nlm.nih.gov/geo/series/GSE75nnn/GSE75731/

SRA Run Selector: https://www.ncbi.nlm.nih.gov/Traces/study/?acc=PRJNA305232

Series Accession: GSE75731 ID: 200075731

12. Genome-wide Annotation, Identification, and Global Transcriptomic Analysis of Regulatory or Small RNA Gene Expression in Staphylococcus aureus

**Excluded – Exclusion based on criteria #5**

(Submitter supplied) In Staphylococcus aureus, hundreds of small regulatory or small RNAs (sRNAs) have been identified, yet this class of molecule remains poorly understood and severely understudied. sRNA genes are typically absent from genome annotation files, and as a consequence, their existence is often overlooked, particularly in global transcriptomic studies. To facilitate improved detection and analysis of sRNAs in S. more...

Organism: Staphylococcus aureus

Type: Expression profiling by high throughput sequencing

Platform: GPL19476 4 Samples

FTP download: GEO (XLS) ftp://ftp.ncbi.nlm.nih.gov/geo/series/GSE74nnn/GSE74936/

SRA Run Selector: https://www.ncbi.nlm.nih.gov/Traces/study/?acc=PRJNA301968

Series Accession: GSE74936 ID: 200074936

13. Investigating the sRNA and mRNA transcriptional response to antibiotics in methicillin-resistant Staphylococcus aureus using Illumina RNAseq

**Excluded – Exclusion based on criteria** **#5**

(Submitter supplied) Coordinated protein-coding sequence transcriptional responses of Staphylococcus aureus to antimicrobial exposure are well described but little is known of the role of bacterial non-coding, small RNAs (sRNAs) in these responses. Here we used RNAseq to investigate the sRNA response of the epidemic multiresistant hospital ST239 S. Aureus strain JKD6009 and its vancomycin-intermediate clinical derivative, JKD6008, after exposure to four antibiotics representing the major classes of antimicrobials used to treat methicillin-resistant S. more...

Organism: Staphylococcus aureus

Type: Expression profiling by high throughput sequencing; Non-coding RNA profiling by high throughput sequencing

Platform: GPL16057 40 Samples

FTP download: GEO (CSV, TXT) ftp://ftp.ncbi.nlm.nih.gov/geo/series/GSE40nnn/GSE40864/

SRA Run Selector: https://www.ncbi.nlm.nih.gov/Traces/study/?acc=PRJNA175138

Series Accession: GSE40864 ID: 200040864

14. Saureus_HG003_RNAseq

**Excluded – Data set already accounted for in results above**

Organism: Staphylococcus aureus

Source name: Bacteria

Platform: GPL16057 Series: GSE104971

FTP download: GEO (GFF) ftp://ftp.ncbi.nlm.nih.gov/geo/samples/GSM2811nnn/GSM2811258/

SRA Run Selector: https://www.ncbi.nlm.nih.gov/Traces/study/?acc=SRX3283115

Sample Accession: GSM2811258 ID: 302811258

15. CP-treated [WT_CP_3]

**Excluded – Data set already accounted for in results above**

Organism: Staphylococcus aureus

Source name: Exponentially grown cells

Platform: GPL19006 Series: GSE99432

FTP download:

SRA Run Selector: https://www.ncbi.nlm.nih.gov/Traces/study/?acc=SRX2868749

Sample Accession: GSM2643955 ID: 302643955

16. CP-treated [WT_CP_2]

**Excluded – Data set already accounted for in results above**

Organism: Staphylococcus aureus

Source name: Exponentially grown cells

Platform: GPL19006 Series: GSE99432

FTP download:

SRA Run Selector: https://www.ncbi.nlm.nih.gov/Traces/study/?acc=SRX2868748

Sample Accession: GSM2643954 ID: 302643954

17. CP-treated [WT_CP_1]

**Excluded – Data set already accounted for in results above**

Organism: Staphylococcus aureus

Source name: Exponentially grown cells

Platform: GPL19006 Series: GSE99432

FTP download:

SRA Run Selector: https://www.ncbi.nlm.nih.gov/Traces/study/?acc=SRX2868747

Sample Accession: GSM2643953 ID: 302643953

18. WT unstressed (CP control) [WT_untreated_3]

**Excluded – Data set already accounted for in results above**

Organism: Staphylococcus aureus

Source name: Exponentially grown cells

Platform: GPL19006 Series: GSE99432

FTP download:

SRA Run Selector: https://www.ncbi.nlm.nih.gov/Traces/study/?acc=SRX2868746

Sample Accession: GSM2643952 ID: 302643952

19. WT unstressed (CP control) [WT_untreated_2]

**Excluded – Data set already accounted for in results above**

Organism: Staphylococcus aureus

Source name: Exponentially grown cells

Platform: GPL19006 Series: GSE99432

FTP download:

SRA Run Selector: https://www.ncbi.nlm.nih.gov/Traces/study/?acc=SRX2868745

Sample Accession: GSM2643951 ID: 302643951

20. WT unstressed (CP control) [WT_untreated_1]

**Excluded – Data set already accounted for in results above**

Organism: Staphylococcus aureus

Source name: Exponentially grown cells

Platform: GPL19006 Series: GSE99432

FTP download:

SRA Run Selector: https://www.ncbi.nlm.nih.gov/Traces/study/?acc=SRX2868744

Sample Accession: GSM2643950 ID: 302643950

21. MW2 RPMI liquid 37C Bioreplicate2 mRNA-seq

**Excluded – Data set already accounted for in results above**

Organism: Staphylococcus aureus

Source name: exponentially growing culture

Platform: GPL17452 Series: GSE85110

FTP download:

SRA Run Selector: https://www.ncbi.nlm.nih.gov/Traces/study/?acc=SRX1995473

Sample Accession: GSM2257876 ID: 302257876

22. MW2 RPMI liquid 37C Bioreplicate1 mRNA-seq

**Excluded – Data set already accounted for in results above**

Organism: Staphylococcus aureus

Source name: exponentially growing culture

Platform: GPL17452 Series: GSE85110

FTP download:

SRA Run Selector: https://www.ncbi.nlm.nih.gov/Traces/study/?acc=SRX1995472

Sample Accession: GSM2257875 ID: 302257875

23. Newman wild-type rep2_RNASeq

**Excluded – Data set already accounted for in results above**

Organism: Staphylococcus aureus subsp. aureus str. Newman

Source name: S. aureus cells

Platform: GPL20326 Series: GSE75731

FTP download:

SRA Run Selector: https://www.ncbi.nlm.nih.gov/Traces/study/?acc=SRX1466096

Sample Accession: GSM1964856 ID: 301964856

24. Newman wild-type rep1_RNASeq

**Excluded – Data set already accounted for in results above**

Organism: Staphylococcus aureus subsp. aureus str. Newman

Source name: S. aureus cells

Platform: GPL20326 Series: GSE75731

FTP download:

SRA Run Selector: https://www.ncbi.nlm.nih.gov/Traces/study/?acc=SRX1466095

Sample Accession: GSM1964855 ID: 301964855

25. DHBP rep2_RNASeq

**Excluded – Data set already accounted for in results above**

Organism: Staphylococcus aureus subsp. aureus str. Newman

Source name: S. aureus cells

Platform: GPL20326 Series: GSE75731

FTP download:

SRA Run Selector: https://www.ncbi.nlm.nih.gov/Traces/study/?acc=SRX1466102

Sample Accession: GSM1964862 ID: 301964862

26. DHBP rep1_RNASeq

**Excluded – Data set already accounted for in results above**

Organism: Staphylococcus aureus subsp. aureus str. Newman

Source name: S. aureus cells

Platform: GPL20326 Series: GSE75731

FTP download:

SRA Run Selector: https://www.ncbi.nlm.nih.gov/Traces/study/?acc=SRX1466101

Sample Accession: GSM1964861 ID: 301964861

27. WalKV149A rep2_RNASeq

**Excluded – Data set already accounted for in results above**

Organism: Staphylococcus aureus subsp. aureus str. Newman

Source name: S. aureus cells

Platform: GPL20326 Series: GSE75731

FTP download:

SRA Run Selector: https://www.ncbi.nlm.nih.gov/Traces/study/?acc=SRX1466100

Sample Accession: GSM1964860 ID: 301964860

28. WalKV149A rep1_RNASeq

**Excluded – Data set already accounted for in results above**

Organism: Staphylococcus aureus subsp. aureus str. Newman

Source name: S. aureus cells

Platform: GPL20326 Series: GSE75731

FTP download:

SRA Run Selector: https://www.ncbi.nlm.nih.gov/Traces/study/?acc=SRX1466099

Sample Accession: GSM1964859 ID: 301964859

29. WalKD119A rep2_RNASeq

**Excluded – Data set already accounted for in results above**

Organism: Staphylococcus aureus subsp. aureus str. Newman

Source name: S. aureus cells

Platform: GPL20326 Series: GSE75731

FTP download:

SRA Run Selector: https://www.ncbi.nlm.nih.gov/Traces/study/?acc=SRX1466098

Sample Accession: GSM1964858 ID: 301964858

30. WalKD119A rep1_RNASeq

**Excluded – Data set already accounted for in results above**

Organism: Staphylococcus aureus subsp. aureus str. Newman

Source name: S. aureus cells

Platform: GPL20326 Series: GSE75731

FTP download:

SRA Run Selector: https://www.ncbi.nlm.nih.gov/Traces/study/?acc=SRX1466097

Sample Accession: GSM1964857 ID: 301964857
