# Supplementary material for: Reading between the Lines: Utilizing RNA-Seq Data for Global Analysis of sRNAs in Staphylococcus aureus
Source: mSphere. 2020 Jul 29;5(4):e00439-20. doi: 10.1128/mSphere.00439-20 (PMC7392542; doi:10.1128/mSphere.00439-20)
Supplement: TEXT S3 [file mSphere.00439-20-s0003.docx]

Search term “*Staphylococcus aureus* RNA-seq”

Date of search Oct 31 2018

1. Next Generation Sequencing Facilitates Quantitative Analysis of S. aureus subsp. aureus ST398 and ST239 transcriptomes

**Excluded – Exclusion based on criteria #4**

(Submitter supplied) Purpose: Next-generation sequencing (NGS) has revolutionized systems-based analysis of cellular pathways. The goal of this study is to investigate the significantly different pathways and genes between ST398 and ST239. Methods: mRNA profiles of ST398 and ST239 at mid-logarithmic growth phase (4h) were generated by deep sequencing, respectively in quadruplicate and duplicate samples, using the Hiseq2000 (Illumina, CA) sequencer. more...

Organism: Staphylococcus aureus subsp. aureus ST398; Staphylococcus aureus CN79

Type: Expression profiling by high throughput sequencing

Platforms: GPL20753 GPL20754 6 Samples

FTP download: GEO (TXT) ftp://ftp.ncbi.nlm.nih.gov/geo/series/GSE71nnn/GSE71516/

SRA Run Selector: https://www.ncbi.nlm.nih.gov/Traces/study/?acc=PRJNA291418

Series Accession: GSE71516 ID: 200071516

2. The conserved regulatory RNA RsaE down-regulates the arginine degradation pathway in Staphylococcus aureus [ssRNA-Seq]

**Included as Study #007**

(Submitter supplied) Bacterial regulatory RNAs (sRNA) generally act by base-pairing with target mRNAs. While identification of sRNA targets is the essential step in sRNA characterization, it remains a stumbling block in most studies. To study sRNA-regulated networks in the major human pathogen Staphylococcus aureus, we used a RNA-RNA interactome screening method for identifying sRNA targets based on synthetic sRNAs that are used in vitro as bait to trap their corresponding targets. more...

Organism: Staphylococcus aureus

Type: Expression profiling by high throughput sequencing

Platform: GPL24214 10 Samples

FTP download: GEO (TXT) ftp://ftp.ncbi.nlm.nih.gov/geo/series/GSE106nnn/GSE106456/

SRA Run Selector: https://www.ncbi.nlm.nih.gov/Traces/study/?acc=PRJNA416921

Series Accession: GSE106456 ID: 200106456

3. The conserved regulatory RNA RsaE down-regulates the arginine degradation pathway in Staphylococcus aureus [Hybrid-Trap-seq]

**Excluded – Exclusion based on criteria #3**

(Submitter supplied) Bacterial regulatory RNAs (sRNA) generally act by base-pairing with target mRNAs. While identification of sRNA targets is the essential step in sRNA characterization, it remains a stumbling block in most studies. To study sRNA-regulated networks in the major human pathogen Staphylococcus aureus, we used a RNA-RNA interactome screening method for identifying sRNA targets based on synthetic sRNAs that are used in vitro as bait to trap their corresponding targets. more...

Organism: Staphylococcus aureus

Type: Expression profiling by high throughput sequencing; Other

Platform: GPL16057 4 Samples

FTP download: GEO (CSV, GFF) ftp://ftp.ncbi.nlm.nih.gov/geo/series/GSE106nnn/GSE106327/

SRA Run Selector: https://www.ncbi.nlm.nih.gov/Traces/study/?acc=PRJNA416319

Series Accession: GSE106327 ID: 200106327

4. Changes in relative transcript amounts caused by treatment of streptozotocin and floxuridine in S.aureus USA300

**Included as Study #008**

(Submitter supplied) Staphylococcus aureus is an important human pathogen causing skin infection and many serious diseases such as pneumonia, sepsis, and toxic shock syndrome. We identified two anti-cancer drugs, streptozotocin (STZ) and floxuridine (FU), as promising lead compounds for further optimization into effective anti-virulence drugs against S. aureus infections. To understand the molecular mechanism of the in vivo efficacy of STZ and FU, we carried out RNA-seq. more...

Organism: Staphylococcus aureus

Type: Expression profiling by high throughput sequencing

Platform: GPL24034 9 Samples

FTP download: GEO (TXT) ftp://ftp.ncbi.nlm.nih.gov/geo/series/GSE104nnn/GSE104069/

SRA Run Selector: https://www.ncbi.nlm.nih.gov/Traces/study/?acc=PRJNA408150

Series Accession: GSE104069 ID: 200104069

5. Effects of fosfomycin on biofilm of a clinical Staphylococcus aureus isolated from osteoarticular infection by transcriptomal approach

**Included as Study #009**

(Submitter supplied) The aim of the present study was to investigate the mechanisms by which fosfomycin restrains biofilm formation and affects a 24h-old biofilm of S. aureus. RNA sequencing (RNA-Seq) technology was used to compare the transcriptomes of S. aureus biofilms formed or treated with sublethal concentrations of fosfomycin.

Organism: Staphylococcus aureus

Type: Expression profiling by high throughput sequencing

Platform: GPL19006 12 Samples

FTP download: GEO (XLS) ftp://ftp.ncbi.nlm.nih.gov/geo/series/GSE83nnn/GSE83269/

SRA Run Selector: https://www.ncbi.nlm.nih.gov/Traces/study/?acc=PRJNA325459

Series Accession: GSE83269 ID: 200083269

6. Linoleic Acid Stimulation of WT Staphylococcus aureus USA300 NRS384

**Included as Study #010**

(Submitter supplied) Methicillin-resistant Staphylococcus aureus (MRSA) infections result in more than 200,000 hospitalizations and 10,000 deaths in the United States each year and remain an important medical challenge. A key factor of S. aureus pathogenesis is the production of virulence proteins that are secreted into the extracellular matrix damaging host tissues and forming abscesses that may serve as replicative niches for the bacteria. more...

Organism: Staphylococcus aureus USA300-0114

Type: Expression profiling by high throughput sequencing

Platform: GPL23795 6 Samples

FTP download: GEO (TAB, TXT) ftp://ftp.ncbi.nlm.nih.gov/geo/series/GSE101nnn/GSE101580/

SRA Run Selector: https://www.ncbi.nlm.nih.gov/Traces/study/?acc=PRJNA397197

Series Accession: GSE101580 ID: 200101580

7. RNA-seq of Wildtype Staphylococcus aureus USA300 NRS384

**Included as Study #011**

(Submitter supplied) Methicillin-resistant Staphylococcus aureus (MRSA) infections result in more than 200,000 hospitalizations and 10,000 deaths in the United States each year and remain an important medical challenge. To better understand the transcriptome of Staphylococcus aureus USA300 NRS384, a community-acquired MRSA strain, we have conducted an RNA-Seq experiment on WT samples.

Organism: Staphylococcus aureus USA300-0114

Type: Expression profiling by high throughput sequencing

Platform: GPL23795 6 Samples

FTP download: GEO (TAB, TXT) ftp://ftp.ncbi.nlm.nih.gov/geo/series/GSE102nnn/GSE102279/

SRA Run Selector: https://www.ncbi.nlm.nih.gov/Traces/study/?acc=PRJNA397207

Series Accession: GSE102279 ID: 200102279

8. RNA-Seq based comparison of Staphylococcus aureus strains resistent and sensitive to MT02

**Included as Study #012**

(Submitter supplied) A MT02 sensitive and resistant strain of Staphylococcus LAC USA300 JE2 were exposed to MT02. From those cell RNA was extracted and anylsed by RNA-Seq

Organism: Staphylococcus aureus

Type: Expression profiling by high throughput sequencing

Platform: GPL16057 4 Samples

FTP download: GEO (WIG) ftp://ftp.ncbi.nlm.nih.gov/geo/series/GSE87nnn/GSE87572/

SRA Run Selector: https://www.ncbi.nlm.nih.gov/Traces/study/?acc=PRJNA345213

Series Accession: GSE87572 ID: 200087572

9. The Staphylococcus aureus α-Acetolactate Synthase ALS Confers Resistance to Nitrosative Stress

**Included as Study #013**

(Submitter supplied) Staphylococcus aureus is a worldwide pathogen that colonizes the human nasal cavity and is a major cause of respiratory and cutaneous infections. In the nasal cavity, S. aureus thrives with high concentrations of nitric oxide (NO) produced by the innate immune effectors and has available for growth slow metabolizing free hexoses, such as galactose. Here, we have used deep sequencing transcriptomic analysis (RNA-Seq) and 1H-NMR to uncover how S. more...

Organism: Staphylococcus aureus subsp. aureus USA300_FPR3757

Type: Expression profiling by high throughput sequencing

Platform: GPL23534 4 Samples

FTP download: GEO (TXT) ftp://ftp.ncbi.nlm.nih.gov/geo/series/GSE99nnn/GSE99563/

SRA Run Selector: https://www.ncbi.nlm.nih.gov/Traces/study/?acc=PRJNA388956

Series Accession: GSE99563 ID: 200099563

10. Response to low-level phage predation in Staphylococcus aureus biofilms

**Included as Study #014**

(Submitter supplied) An important lesson from the war on pathogenic bacteria has been the need to understand the physiological responses and evolution of natural microbial communities. Bacterial populations in the environment are generally forming biofilms subject to some level of phage predation. These multicellular communities are notoriously resistant to antimicrobials and, consequently, very difficult to eradicate. This has sparked the search for new therapeutic alternatives, including phage therapy. This study demonstrates that S. aureus biofilms formed in the presence of a non-lethal dose of phage phiIPLA-RODI exhibit a unique physiological state that could potentially benefit both the host and the predator. Thus, biofilms formed under phage pressure are thicker and have a greater DNA content. Also, the virus-infected biofilm displayed major transcriptional differences compared to an untreated control. Significantly, RNA-seq data revealed activation of the stringent response, which could slow down the advance of the bacteriophage within the biofilm. The end result would be an equilibrium that would help bacterial cells to withstand environmental challenges, while maintaining a reservoir of sensitive bacterial cells available to the phage upon reactivation of the dormant carrier population.

Organism: Staphylococcus aureus

Type: Expression profiling by high throughput sequencing

Platform: GPL17452 6 Samples

FTP download: GEO (TXT) ftp://ftp.ncbi.nlm.nih.gov/geo/series/GSE87nnn/GSE87706/

SRA Run Selector: https://www.ncbi.nlm.nih.gov/Traces/study/?acc=PRJNA345608

Series Accession: GSE87706 ID: 200087706

11. Phenotype and RNA-Seq-Based Transcriptome Profiling of Staphylococcus aureus biofilms in response to Tea tree oil

**Included as Study #015**

(Submitter supplied) RNA sequencing (RNA-Seq) was used in our study to elucidate the mechanism of Tea tree oil (TTO) as a potential antibacterial agent to evaluate differentially expressed genes and functional network analysis in S. aureus ATCC 29213 biofilms.

Organism: Staphylococcus aureus

Type: Expression profiling by high throughput sequencing

Platform: GPL19006 4 Samples

FTP download: GEO (XLS) ftp://ftp.ncbi.nlm.nih.gov/geo/series/GSE85nnn/GSE85787/

SRA Run Selector: https://www.ncbi.nlm.nih.gov/Traces/study/?acc=PRJNA339391

Series Accession: GSE85787 ID: 200085787

12. Characterization of the LFR Genomic Islet in Staphylococcus aureus CC30

**Included as Study #002**

(Submitter supplied) We report the application of RNA-Seq to assess transcriptional profiles of S. aureus CC30 strains with allelic replacements (knockouts) of two genes in the LFR genomic islet: 1) fatty acid desaturase (fad) and 2) a MocR regulator. The overall results of our study suggest that the LFR islet enhances metabolic plasticity of the CC30 lineage which contributes to increased colonization, survival and persistence in the host.

Organism: Staphylococcus aureus

Type: Expression profiling by high throughput sequencing

Platform: GPL19006 14 Samples

FTP download: GEO (TXT) ftp://ftp.ncbi.nlm.nih.gov/geo/series/GSE83nnn/GSE83995/

SRA Run Selector: https://www.ncbi.nlm.nih.gov/Traces/study/?acc=PRJNA327760

Series Accession: GSE83995 ID: 200083995

13. The mutual effect of Staphylococcus epidermidis and Staphylococcus aureus in dual species biofilms as revealed by RNA-Seq

**Excluded – Exclusion based on criteria #1**

(Submitter supplied) We examined the differential gene expression of Staphylococcus epidermidis and Staphylococcus epidermidis in dual species biofilms. Therefore, we performed RNA-Seq on single and dual species biofilms and we compared the gene expression levels in dual species biofilms to those in single species biofilms.

Organism: Staphylococcus aureus; Staphylococcus epidermidis

Type: Expression profiling by high throughput sequencing

Platforms: GPL21653 GPL17452 GPL21652 9 Samples

FTP download: GEO (XLSX) ftp://ftp.ncbi.nlm.nih.gov/geo/series/GSE79nnn/GSE79575/

SRA Run Selector: https://www.ncbi.nlm.nih.gov/Traces/study/?acc=PRJNA316215

Series Accession: GSE79575 ID: 200079575

14. A master virulence regulator of S. aureus inactivated during carriage in man

**Included as Study #005**

(Submitter supplied) It remains unclear how the bacterial populations which colonise many healthy humans occasionally give rise to severe disease. Staphylococcus aureus represents one such population. Here we describe mutations in invasive S. aureus which arose during human colonisation and inactivated rsp, a transcription factor. RNA-Seq was used to generate mRNA profiles of genes controlled by rsp.

Organism: Staphylococcus aureus

Type: Expression profiling by high throughput sequencing

Platform: GPL17452 12 Samples

FTP download: GEO (TXT) ftp://ftp.ncbi.nlm.nih.gov/geo/series/GSE67nnn/GSE67448/

SRA Run Selector: https://www.ncbi.nlm.nih.gov/Traces/study/?acc=PRJNA279958

Series Accession: GSE67448 ID: 200067448

15. Comparative virulence studies and transcriptome analysis of Staphylococcus aureus strains isolated from animals

**Included as Study #016**

(Submitter supplied) Staphylococcus aureus can infect a wide range of animals and pose as a serious threat to public health by transferring via animals or animal-derived food stuff. Even more importantly, multiple drug resistance development in the bacteria has resulted in therapeutic failure of a number of antibiotics. Therefore by realizing the need of time, this study was designed to investigate the underlying mechanisms of virulence and resistance in S. more...

Organism: Staphylococcus aureus

Type: Expression profiling by high throughput sequencing

Platform: GPL19006 4 Samples

FTP download: GEO (TXT) ftp://ftp.ncbi.nlm.nih.gov/geo/series/GSE78nnn/GSE78764/

SRA Run Selector: https://www.ncbi.nlm.nih.gov/Traces/study/?acc=PRJNA313454

Series Accession: GSE78764 ID: 200078764

16. RNA-Seq-mediated transcriptome analysis of Staphylococcus aureus Newman wild-type, walKD119A, walKV149A and DHBP-treated wild-type strains

**Included as Study #006 (repeat from File Search 1)**

(Submitter supplied) We reported the RNA-Seq results of Staphylococcus aureus Newman wild-type, walKD119A, walKV149A and DHBP-treated wild-type strains. We found that mutations of the potential signal transduction residues of WalK attenuate the activity of walKR two-component system, whereas DHBP supplementation activates this two-component system.

Organism: Staphylococcus aureus subsp. aureus str. Newman

Type: Expression profiling by high throughput sequencing

Platform: GPL20326 8 Samples

FTP download: GEO (TXT) ftp://ftp.ncbi.nlm.nih.gov/geo/series/GSE75nnn/GSE75731/

SRA Run Selector: https://www.ncbi.nlm.nih.gov/Traces/study/?acc=PRJNA305232

Series Accession: GSE75731 ID: 200075731

17. Next Generation Sequencing Facilitates Quantitative Analysis of Wild Type and △Rsp MRSA Transcriptomes

**Included as Study #018**

(Submitter supplied) Purpose: Next-generation sequencing (NGS) has revolutionized systems-based analysis of gene regulon. The goal of this study is to investigate the genes regulated by Rsp in MRSA BD02-25 Methods: mRNA profiles of wild-type (WT) and Rsp knockout (△Rsp) MRSA at mid-logarithmic growth phase (4h) were generated by deep sequencing, respectively in duplicate samples, using the Hiseq2000 (Illumina, CA) sequencer. more...

Organism: Staphylococcus aureus

Type: Expression profiling by high throughput sequencing

Platform: GPL17452 4 Samples

FTP download: GEO (TXT) ftp://ftp.ncbi.nlm.nih.gov/geo/series/GSE67nnn/GSE67344/

SRA Run Selector: https://www.ncbi.nlm.nih.gov/Traces/study/?acc=PRJNA279553

Series Accession: GSE67344 ID: 200067344

18. RNA-SEQ Reveals Changes in the Staphylococcus aureus Transcriptome following blue light illumination

**Included as Study #019**

(Submitter supplied) Purpose: In an effort to better understand the mechanism of blue light inhibition in Staphylococcus aureus, a whole transcriptome analysis of S. aureus isolate BUSA2288 was performed using RNA-seq to analyze the differential gene expression in response to blue light exposure. Methods: RNA was extracted from S. aureus cultures pooled from 24 one ml well samples illuminated with a dose of 250 J/cm2 of 465 nm blue light, and from control cultures grown in the dark. more...

Organism: Staphylococcus aureus

Type: Non-coding RNA profiling by high throughput sequencing

Platform: GPL18481 4 Samples

FTP download: GEO (TXT) ftp://ftp.ncbi.nlm.nih.gov/geo/series/GSE62nnn/GSE62055/

SRA Run Selector: https://www.ncbi.nlm.nih.gov/Traces/study/?acc=PRJNA263102

Series Accession: GSE62055 ID: 200062055

19. Decay-initiating endoribonucleolytic cleavage by RNase Y is kept under tight control via sequence preference and sub-cellular localization

**Included as Study #17**

(Submitter supplied) Bacteria depend on efficient RNA turnover to rapidly alter gene expression, essentially for responding to changing conditions. Nevertheless, remarkably few details are known about the rate-limiting steps in targeting and decay of RNA. The membrane-anchored endoribonuclease RNase Y is a virulence factor in Gram- positive pathogens. We have obtained a global picture of RNase Y sequence specificity using RNA-seq and the novel transcriptome-wide EMOTE method. more...

Organism: Staphylococcus aureus

Type: Expression profiling by high throughput sequencing; Other

Platform: GPL19006 24 Samples

FTP download: GEO (TXT) ftp://ftp.ncbi.nlm.nih.gov/geo/series/GSE68nnn/GSE68811/

SRA Run Selector: https://www.ncbi.nlm.nih.gov/Traces/study/?acc=PRJNA283825

Series Accession: GSE68811 ID: 200068811

20. The C-terminal region of the RNA helicase CshA is required for the interaction with the degradosome and turnover of bulk RNA in the opportunistic pathogen Staphylococcus aureus.

**Included as Study #020**

(Submitter supplied) Staphylococcus aureus is a versatile opportunistic pathogen that adapts readily to a variety of different growth conditions. This adaptation requires a rapid regulation of gene expression including the control of mRNA abundance. The CshA DEAD-box RNA helicase was previously shown to be required for efficient turnover of the agr quorum sensing mRNA. Here we show by transcriptome-wide RNA sequencing and microarray analyses that CshA is required for the degradation of bulk mRNA. more...

Organism: Staphylococcus aureus

Type: Expression profiling by high throughput sequencing

Platform: GPL19006 24 Samples

FTP download: GEO (TXT) ftp://ftp.ncbi.nlm.nih.gov/geo/series/GSE68nnn/GSE68772/

SRA Run Selector: https://www.ncbi.nlm.nih.gov/Traces/study/?acc=PRJNA283834

Series Accession: GSE68772 ID: 200068772

21. SaeRS-dependent inhibition of biofilm formation in Staphylococcus aureus Newman

**Included as Study #021**

(Submitter supplied) The SaeRS two-component regulatory system of Staphylococcus aureus is known to affect the expression of many genes. The SaeS protein is the histidine kinase responsible for phosphorylation of the response regulator SaeR. In S. aureus Newman, the sae system is constitutively expressed due to a point mutation in saeS, relative to other S. aureus strains, which results in substitution of proline for leucine at amino acid 18. more...

Organism: Staphylococcus aureus

Type: Expression profiling by high throughput sequencing

Platform: GPL18481 10 Samples

FTP download: GEO (TXT) ftp://ftp.ncbi.nlm.nih.gov/geo/series/GSE65nnn/GSE65827/

SRA Run Selector: https://www.ncbi.nlm.nih.gov/Traces/study/?acc=PRJNA275146

Series Accession: GSE65827 ID: 200065827

22. Comparative genomics of Staphylococcus aureus and the application of a “pan-genome” for assigning RNA-Seq transcript reads from divergent strains and in vivo human nasal samples

**Excluded – Exclusion based on criteria** **#2**

(Submitter supplied) in vitro comparison between two MRSA grown in rich (BHI) and poor media (SNM), compared with the nasal metatranscriptome reads of S. aureus.

Organism: Staphylococcus aureus

Type: Expression profiling by high throughput sequencing

Platforms: GPL18481 GPL16057 7 Samples

FTP download: GEO (TXT) ftp://ftp.ncbi.nlm.nih.gov/geo/series/GSE56nnn/GSE56294/

SRA Run Selector: https://www.ncbi.nlm.nih.gov/Traces/study/?acc=PRJNA242859

Series Accession: GSE56294 ID: 200056294

23. Comparative transcriptome of S. aureus strains

**Excluded – Exclusion based on criteria** **#2**

(Submitter supplied) We report the transcriptome profiles (RNA seq) of three different co-isolates of S.aureus that have been identified and isolated in both laboratory and infective scenarios. The transcriptome profiles were generated by deep sequencing and transcript levels assessed. For this the raw reads underwent quality-trimming (using the FastX suite), polyA-clipping, size filtering, mapping to the reference genome, coverage calculation, gene wise expression quantification followed by differential gene expression analysis (all done by the tool "READemption" (Förstner et al., unpublished) using "segemehl" (Hoffmann et al., 2009) and "DESeq" (Anders et al., 2010). more...

Organism: Staphylococcus aureus subsp. aureus USA300

Type: Expression profiling by high throughput sequencing

Platform: GPL17544 3 Samples

FTP download: GEO (WIG) ftp://ftp.ncbi.nlm.nih.gov/geo/series/GSE49nnn/GSE49636/

SRA Run Selector: https://www.ncbi.nlm.nih.gov/Traces/study/?acc=PRJNA214549

Series Accession: GSE49636 ID: 200049636

24. Potential Influence of Staphylococcus aureus Clonal Complex 30 Genotype and Transcriptome on Hematogenous Infections

**Excluded – Exclusion based on criteria** **#2**

(Submitter supplied) We report the application of RNA-Seq to assess genomic transcriptional profiles of S. aureus clinical isolates from Clonal Complex 30 and related genetic backgrounds. In this study, CC30 isolates were shown to be significantly less virulent than other CCs in two in vivo sepsis models. The association of CC30 with adhesin-based complications in humans but not sepsis-induced mortality in animal models may be due in part to its unique genomic transcriptional profile and suggests that specific genotypes of S. more...

Organism: Staphylococcus aureus

Type: Expression profiling by high throughput sequencing

Platform: GPL19006 32 Samples

FTP download: GEO (TXT) ftp://ftp.ncbi.nlm.nih.gov/geo/series/GSE59nnn/GSE59851/

SRA Run Selector: https://www.ncbi.nlm.nih.gov/Traces/study/?acc=PRJNA255909

Series Accession: GSE59851 ID: 200059851

25. Steady-state hydrogen peroxide induces glycolysis via metabolic reroute in P. aeruginosa and S. aureus

**Included as Study #022**

(Submitter supplied) GAPDHs from human pathogens S. aureus and P. aeruginosa can be readily inhibited by ROS-mediated direct oxidation of their catalytic active cysteines. Because of the rapid degradation of H2O2 by bacterial catalase, only steady-state but not one-dose treatment of H2O2 induces rapid metabolic reroute from glycolysis to pentose phosphate pathway (PPP). We conducted RNA-seq analyses to globally profile the bacterial transcriptomes in response to a steady level of H2O2, which reveals profound transcriptional changes including the induced expression of glycolytic genes in both bacteria. more...

Organism: Staphylococcus aureus subsp. aureus str. Newman; Pseudomonas aeruginosa MPAO1/P1

Type: Expression profiling by high throughput sequencing

Platforms: GPL18362 GPL18363 8 Samples

FTP download: GEO (DIFF) ftp://ftp.ncbi.nlm.nih.gov/geo/series/GSE55nnn/GSE55528/

SRA Run Selector: https://www.ncbi.nlm.nih.gov/Traces/study/?acc=PRJNA239877

Series Accession: GSE55528 ID: 200055528

26. RNA-seq reveals differential gene expression in Staphylococcus aureus at single-nucleotide resolution

**Excluded – Exclusion based on criteria** **#2**

(Submitter supplied) Staphylococcus aureus is a gram-positive cocci and an important human commensal bacteria and pathogen. S. aureus infections are increasingly difficult to treat because of the emergence of highly resistant MRSA (Methicillin-resistant S. aureus) strains. Here we present a method to study differential gene expression in S. aureus using high-throughput RNA-sequencing (RNA-seq). We use RNA-seq to examine the differential gene expression in S. more...

Organism: Staphylococcus aureus

Type: Expression profiling by high throughput sequencing

Platform: GPL17452 3 Samples

FTP download: GEO (WIG) ftp://ftp.ncbi.nlm.nih.gov/geo/series/GSE48nnn/GSE48896/

SRA Run Selector: https://www.ncbi.nlm.nih.gov/Traces/study/?acc=PRJNA212142

Series Accession: GSE48896 ID: 200048896

27. WT 2C 10 uM Linoleic Acid

**Excluded – Data set already accounted for in results above**

Organism: Staphylococcus aureus USA300-0114

Source name: S. aureus USA300 NRS384 cultures

Platform: GPL23795 Series: GSE101580

FTP download: GEO (TAB) ftp://ftp.ncbi.nlm.nih.gov/geo/samples/GSM2706nnn/GSM2706364/

SRA Run Selector: https://www.ncbi.nlm.nih.gov/Traces/study/?acc=SRX3062755

Sample Accession: GSM2706364 ID: 302706364

28. WT 2B 10 uM Linoleic Acid

**Excluded – Data set already accounted for in results above**

Organism: Staphylococcus aureus USA300-0114

Source name: S. aureus USA300 NRS384 cultures

Platform: GPL23795 Series: GSE101580

FTP download: GEO (TAB) ftp://ftp.ncbi.nlm.nih.gov/geo/samples/GSM2706nnn/GSM2706363/

SRA Run Selector: https://www.ncbi.nlm.nih.gov/Traces/study/?acc=SRX3062754

Sample Accession: GSM2706363 ID: 302706363

29. WT 2A 10 uM Linoleic Acid

**Excluded – Data set already accounted for in results above**

Organism: Staphylococcus aureus USA300-0114

Source name: S. aureus USA300 NRS384 cultures

Platform: GPL23795 Series: GSE101580

FTP download: GEO (TAB) ftp://ftp.ncbi.nlm.nih.gov/geo/samples/GSM2706nnn/GSM2706362/

SRA Run Selector: https://www.ncbi.nlm.nih.gov/Traces/study/?acc=SRX3062753

Sample Accession: GSM2706362 ID: 302706362

30. WT 1C No treatment

**Excluded – Data set already accounted for in results above**

Organism: Staphylococcus aureus USA300-0114

Source name: S. aureus USA300 NRS384 cultures

Platform: GPL23795 Series: GSE101580

FTP download: GEO (TAB) ftp://ftp.ncbi.nlm.nih.gov/geo/samples/GSM2706nnn/GSM2706361/

SRA Run Selector: https://www.ncbi.nlm.nih.gov/Traces/study/?acc=SRX3062752

Sample Accession: GSM2706361 ID: 302706361

31. WT 1B No treatment

**Excluded – Data set already accounted for in results above**

Organism: Staphylococcus aureus USA300-0114

Source name: S. aureus USA300 NRS384 cultures

Platform: GPL23795 Series: GSE101580

FTP download: GEO (TAB) ftp://ftp.ncbi.nlm.nih.gov/geo/samples/GSM2706nnn/GSM2706360/

SRA Run Selector: https://www.ncbi.nlm.nih.gov/Traces/study/?acc=SRX3062751

Sample Accession: GSM2706360 ID: 302706360

32. WT 1A No treatment

**Excluded – Data set already accounted for in results above**

Organism: Staphylococcus aureus USA300-0114

Source name: S. aureus USA300 NRS384 cultures

Platform: GPL23795 Series: GSE101580

FTP download: GEO (TAB) ftp://ftp.ncbi.nlm.nih.gov/geo/samples/GSM2706nnn/GSM2706359/

SRA Run Selector: https://www.ncbi.nlm.nih.gov/Traces/study/?acc=SRX3062750

Sample Accession: GSM2706359 ID: 302706359

33. SA-HP-2

**Excluded – Data set already accounted for in results above**

Organism: Staphylococcus aureus subsp. aureus str. Newman

Source name: liquid culture in TSB

Platform: GPL18363 Series: GSE55528

FTP download:

SRA Run Selector: https://www.ncbi.nlm.nih.gov/Traces/study/?acc=SRX480726

Sample Accession: GSM1338708 ID: 301338708

34. SA-HP-1

**Excluded – Data set already accounted for in results above**

Organism: Staphylococcus aureus subsp. aureus str. Newman

Source name: liquid culture in TSB

Platform: GPL18363 Series: GSE55528

FTP download:

SRA Run Selector: https://www.ncbi.nlm.nih.gov/Traces/study/?acc=SRX480725

Sample Accession: GSM1338707 ID: 301338707

35. SA-CK-2

**Excluded – Data set already accounted for in results above**

Organism: Staphylococcus aureus subsp. aureus str. Newman

Source name: liquid culture in TSB

Platform: GPL18363 Series: GSE55528

FTP download:

SRA Run Selector: https://www.ncbi.nlm.nih.gov/Traces/study/?acc=SRX480724

Sample Accession: GSM1338706 ID: 301338706

36. SA-CK-1

**Excluded – Data set already accounted for in results above**

Organism: Staphylococcus aureus subsp. aureus str. Newman

Source name: liquid culture in TSB

Platform: GPL18363 Series: GSE55528

FTP download:

SRA Run Selector: https://www.ncbi.nlm.nih.gov/Traces/study/?acc=SRX480723

Sample Accession: GSM1338705 ID: 301338705
